# Exploring the Impact of a Low-Protein High-Carbohydrate Diet in Mature Broodstock of a Glucose-Intolerant Teleost, the Rainbow Trout

**DOI:** 10.3389/fphys.2020.00303

**Published:** 2020-05-15

**Authors:** Thérèse Callet, Huihua Hu, Laurence Larroquet, Anne Surget, Jingwei Liu, Elisabeth Plagnes-Juan, Patrick Maunas, Nicolas Turonnet, Jan Alexander Mennigen, Julien Bobe, Christine Burel, Geneviève Corraze, Stephane Panserat, Lucie Marandel

**Affiliations:** ^1^INRAE, Université de Pau et des Pays de l’Adour, E2S UPPA, NUMEA, Saint-Pée-sur-Nivelle, France; ^2^State Key Laboratory of Freshwater Ecology and Biotechnology, Institute of Hydrobiology, Chinese Academy of Sciences, Wuhan, China; ^3^University of the Chinese Academy of Sciences, Beijing, China; ^4^Department of Biology, University of Ottawa, Ottawa, ON, Canada; ^5^INRAE, LPGP UR1037, Campus de Beaulieu, Rennes, France

**Keywords:** liver, gonad, enzymatic activity, glucose metabolism, pentose phosphate pathway, glycogen, male, female

## Abstract

Sustainable aquaculture production requires a greater reduction in the use of marine-derived ingredients, and one of the most promising solutions today is the augmentation in the proportion of digestible carbohydrates in aquafeed. This challenge is particularly difficult for high trophic level teleost fish as they are considered to be glucose-intolerant (growth delay and persistent postprandial hyperglycemia observed in juveniles fed a diet containing more than 20% of carbohydrates). It was previously suggested that broodstock could potentially use carbohydrates more efficiently than juveniles, probably due to important metabolic changes that occur during gametogenesis. To investigate this hypothesis, 2-year old male and female rainbow trout (*Oncorhynchus mykiss*) were either fed a diet containing no carbohydrates (NC) or a 35%-carbohydrate diet (HC) for an entire reproductive cycle. Zootechnical parameters as well as the activities of enzymes involved in carbohydrate metabolism were measured in livers and gonads. Fish were then reproduced to investigate the effects of such a diet on reproductive performance. Broodstock consumed the HC diet, and in contrast to what is commonly observed in juveniles, they were able to grow normally and they did not display postprandial hyperglycemia. The modulation of their hepatic metabolism, with an augmentation of the glycogenesis, the pentose phosphate pathway and a possible better regulation of gluconeogenesis, may explain their improved ability to use dietary carbohydrates. Although the HC diet did induce precocious maturation, the reproductive performance of fish was not affected, confirming that broodstock are able to reproduce when fed a low-protein high-carbohydrate diet. In conclusion, this exploratory work has shown that broodstock are able to use a diet containing digestible carbohydrates as high as 35% and can then grow and reproduce normally over an entire reproductive cycle for females and at least at the beginning of the cycle for males. These results are highly promising and suggest that dietary carbohydrates can at least partially replace proteins in broodstock aquafeed.

## Introduction

In order to reduce aquaculture’s reliance on wild fish resources and to ensure the sustainability of the salmonid aquaculture, fish meal (FM) and fish oil (FO), the traditional ingredients of aquafeeds, must be replaced by renewable and economically viable alternative products (Naylor et al., 2009). Terrestrial plants are, especially in Europe, considered to be a promising resource (Kamalam et al., 2017). Carbohydrates, which are abundant in plant ingredients, could represent a non-negligible source of energy and their inclusion in aquafeed can help to spare proteins for growth (Hemre et al., 2002), thus providing ecological benefits by diminishing nitrogen excretion. Moreover, the production of plant-derived carbohydrates is economically viable (Prabu et al., 2017). This is particularly true for broodstock breeding, as these animals, given their weight, require a large quantity of expensive feed.

For teleost fish such as rainbow trout, which belong to a high trophic level, the incorporation of plant-derived carbohydrates in aquafeed is limited by the fact that these species are usually considered to be glucose-intolerant (GI) and therefore relatively poor users of dietary carbohydrates (Polakof et al., 2012). When more than 20% of FM is substituted by digestible carbohydrates in the diet, they typically display a decrease in growth and a persistent postprandial hyperglycemia. Different hypotheses have been proposed to explain this phenomena (Kamalam et al., 2017) and among them, is the lack of inhibition in the last step of the hepatic gluconeogenesis (Marandel et al., 2017).

These results were mainly observed on early developmental stages or in juvenile fish. For broodstock, a few studies published in the 1970’s, 80’s and 90’s highlighted changes in carbohydrate metabolism. Evidence supporting an increased utilization of dietary carbohydrates in sexually recrudescent and mature rainbow trout was reported across different tissues and levels of biological organization. At the digestive level, Onishi and Murayama (1970) reported an increased amylase activity in broodstock. For circulating metabolites, Palmer and Ryman (1972) reported a marked improvement in oral glucose tolerance in vitellogenic females that were thought to be due to a reduction of hepatic *de novo* gluconeogenesis (Washburn et al., 1993). Moreover, at the molecular level, changes in the carbohydrate metabolism of the liver and gonads of the rainbow trout clearly demonstrated a higher use of exogenous glucose by the gonads and liver during gametogenesis when fed a commercial diet (Barciela et al., 1993; Soengas et al., 1993a, b). At the endocrine level, studies conducted on migrating salmonids demonstrated a hyperplasia of endocrine pancreas tissue associated with an increase of the number of Langherans islets during sexual development as well as a hypoglycemic stage (Luquet and Watanabe, 1986). Concerning reproductive performance in rainbow trout fed carbohydrate-rich diets, Washburn et al. (1990) showed a significantly higher relative fecundity and survival rate in progeny hatched from female trout, fed a diet in which FM was partially replaced by white flour as a source of dietary carbohydrates compared to fish fed a controlled diet. Moreover, published studies tended to strongly suggest that decreasing the protein content of a diet while maintaining a sufficient energy supply had no adverse effects on female reproduction (Luquet and Watanabe, 1986).

Overall, these latter studies suggested that broodstock have the potential to better tolerate and use carbohydrates during gametogenesis. However, the mechanisms involved have not been clearly identified, especially at a molecular level in the liver and gonads. Moreover, the effect of highly digestible carbohydrates were only investigated in females and were never tested in male broodstock, despite the increasing evidence of paternal effects on progeny phenotypes (Schagdarsurengin and Steger, 2016).

In this context, the present study aimed to give a first overview of phenotype alterations in both mature male and female rainbow trout, fed a diet containing a high amount of highly digestible carbohydrates and a low-protein content. In particular, we evaluated the effect of such a diet on (i) zootechnical parameters, (ii) carbohydrate metabolism in the liver and gonads and (iii) gametogenesis, gamete characteristics and reproductive performance.

## Materials and Methods

### Ethics Approval

Investigations were conducted according to the guiding principles for the use and care of laboratory animals and in compliance with French and European regulations on animal welfare (Décret 2001-464, 29 May 2001 and Directive 2010/63/EU, respectively). This protocol and the project as a whole were approved by the French National Consultative Ethics Committee (reference numbers 201610061056842).

### Experimental Design and Diets

Two isolipidic and isoenergetic experimental diets containing either no carbohydrates (NC, 0% carbohydrates) or a high content of digestible carbohydrates (HC, 35.30% carbohydrates) were prepared in our own facilities (INRA, Donzacq, France) as extruded pellets (BC45 BisVis Clextral^®^, France) (see Table 1). Gelatinized starch was included as the carbohydrate sources, fish meal was used as protein source, and dietary lipids were provided by fish oil and fish meal. The large increase in dietary carbohydrate content in the HC diet was compensated by a decrease in protein content.

**TABLE 1:** Diet composition and fatty acid profile (% total FA).

**(A)**				**(B)**		
	**Diets**				**Diets**	
	**NC**	**HC**			**NC**	**HC**
		
**Ingredients (%)**				Saturated		
Fish meal^a^	77.77	45		14:0	11.55	10.57
Pregelatinized starch^b^	–	37		15:0	1.13	1.42
P 90^c^	2.00	5		16:0	27.34	29.53
Soya meal^d^	12	–		18:0	2.83	4.35
Soy protein concentrate^e^	–	5		*Total saturated*	43.46	46.97
Fish Oil^f^	1.66	3.96		**MUFA**		
Cellulose^g^	2.53	–		16:1	7.66	7.86
Alginate^h^	2	2		18:1	15.39	15.69
Mineral premix^i^	1	1		20:1	4.60	2.06
Vitamin premix^j^	1	1		*Total MUFA*	30.52	26.98
Carophyll pink^k^	0.04	0.04		**PUFA n-6**		
**Proximate composition**				18:2 n-6	3.57	2.14
Dry matter (DM,%)	91.84	96.22		20:4 n-6	0.62	1.22
Crude protein (%DM)	63.89	42.96		*Total PUFA n-6*	4.19	3.36
Crude lipid (%DM)	8.90	9.2		**PUFA n-3**		
Gross energy, kJ/g DM	20.69	20.38		18:3 n-3	1.14	1.18
Ash,% DM	15.93	9.71		18:4 n-3	2.09	1.61
Carbohydrates, % DM	<0.2	34.30		20:4 n-3	0.15	0.00
				20:5 n-3	5.87	6.39
				22:6 n-3	5.84	7.22
				*Total PUFA n-3*	15.09	16.39
				*LC-PUFA n-3*	11.86	13.60
				Sat/PUFA	1.99	2.10
				n3/n6	3.60	4.88

*NC, no-carbohydrate diet; HC, high-carbohydrate diet, DM; dry matter, MUFA; monosaturated fatty acids, PUFA; polyunsaturated fatty acids LC-PUFA; long chain polyunsaturated fatty acids. *^*a*^Sopropeche, Boulogne-sur-Mer, France. ^*b*^Gelatinized corn starch; Roquette, Lestrem, France. ^*c*^Sopropêche. ^*d*^Sudouest aliment. ^*e*^Legouessant. ^*f*^Fish oil; Sopropeche, Boulogne-sur-Mer, France. ^*g*^Upae. ^*h*^Louis François. ^*i*^Supplied the following (/kg diet): calcium carbonate (40% Ca) 2.15 g, magnesium oxide (60% Mg) 1.24 g, ferric citrate 0.2 g, potassium iodide (75% I) 0.4 mg, zinc sulfate (36% Zn) 0.4 g, copper sulfate (25% Cu) 0.3 g, manganese sulfate (33% Mn) 0.3 g, dibasic calcium phosphate (20% Ca, 18% P) 5 g, cobalt sulfate 2 mg, sodium selenite (30% Se) 3 mg, potassium chloride 0.9 g, sodium chloride 0.4 g. Louis François, Marne-la-Vallée, France. ^*j*^Supplied the following (/kg diet): DL-a tocopherol acetate 60 IU, sodium menadione bisulfate 5 mg, retinyl acetate 15,000 IU, DLcholecalciferol 3,000 IU, thiamin 15 mg, riboflavin 30 mg, pyridoxine 15 mg, vit. B12 0.05 mg, nicotinic acid 175 mg, folic acid 500 mg, inositol 1,000 mg, biotin 2.5 mg, calcium panthotenate 50 mg, choline chloride 2000 mg. ^*k*^Astaxanthine, DSM.**

**FIGURE 1:**
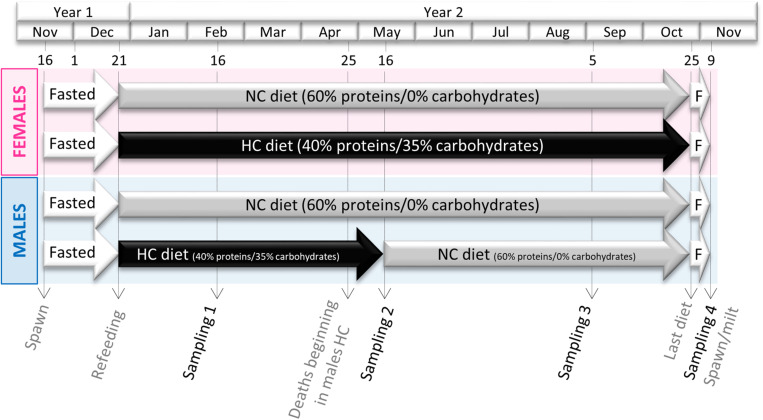
Experimental Design. Both male and female broodstock were fed either a diet containing no carbohydrates (NC) or a high content of digestible carbohydrates (35%, HC) from December of year 1 to the next spawning period at the end of year 2. Because of unexpected mortality in males fed the HC diet at the end of April, surviving males were individually tagged and transferred with other trout fed the NC diet at the beginning of May.

Rainbow trout used in this experiment were autumnal strain and thus their reproduction occurred during winter (November-December). Two-year old males and females (50 of each sex per tank) were distributed in two 8 m^3^ tanks and fed either the NC or the HC diet over a complete reproductive cycle (Figure 1). The feeding trial was thus conducted from the resumption of feeding after the reproduction period (December 2016) to the next reproduction period (November 2017). High mortality induced by a Saprolegnia infection in males fed the HC diet occurred in late April. As we had hypothesized that the HC diet could be a triggering factor in these deaths (see discussion), these males were fed the NC diet from 16 May 2017 until reproduction in order to keep a sufficient amount of fish alive for reproduction.

Fish were sampled in February, May, September and November (sampling 1, 2, 3, and 4 respectively in Figure 1). Males were not sampled in September to ensure enough males were available for reproduction after the deaths, which occurred in May. At each sampling, 8 fish per sex and per diet (except for males fed the HC diet: only 4 were sampled in May) were first anaesthetized in a benzocaine bath at 30 mg⋅L^–1^ and then killed in a benzocaine bath at 60 mg⋅L^–1^. For the first samplings (in February, May and September), samplings were done 6 h after the last meal, while in November, fish were fasted. Blood was drawn from the caudal vein, centrifuged (3,000 g, 5 min) and plasma was recovered and stored at −20°C for future plasma metabolites/steroid analyses. Livers and gonads were dissected, two parts were snap frozen in liquid nitrogen for RNA extraction and enzymatic activities and another part was kept at −20°C for biochemical analysis. The digestive tract (stomach, intestine and peripheral fat) and the remaining whole carcass (without the liver, gonads and digestive tract) were also kept at −20°C for biochemical analyses. For ovaries/mature oocytes, a piece was conserved in physiological serum for dissection, in order to count the number of oocytes and to evaluate their diameters (see below). During the spawning period, spawns from 3 NC females and 2 HC females were cross-fertilized with milts from 4 males from each experimental condition (NC and HC diets). Each of the 40 crossings (Supplementary Figure S1) were closely monitored for survival at eyed stage and hatching, and for the presence of malformations. For each sampling, plasma analysis and oocyte numbers/diameters were determined on the 8 fish per condition whereas RT-qPCR, biochemical compositions and fatty acids compositions were performed on a randomly selected selection of 6 fish.

### Chemical Composition of the Diets

The proximate composition of the diets was analyzed as follows: dry matter was determined after drying to constant mass at 105°C; crude protein (N × 6.25) was determined by the Kjeldahl method after acid digestion; crude lipid was determined by petroleum ether extraction (Soxtherm); gross energy was measured in an adiabatic bomb calorimeter (IKA, Heitersheim Gribeimer, Germany); ash was estimated through incineration in a muffle furnace for 6 h at 600°C.

### Biochemical Composition of Gonads, Livers, Digestive Tract, and Carcasses

Glycogen and glucose content were analyzed on lyophilized organs. Glycogen content was determined by a hydrolysis technique previously described by Good et al. (1933). Each sample was ground in 1 mol⋅l^–1^ HCl (VWR, United States). An aliquot was saved at this stage to measure the free glucose content. After 10 min centrifugation at 10,000 g, free glucose was measured using the Amplite^TM^ Fluorimetric Glucose Quantitation Kit (AAT Bioquest^®^, Inc., United States) according to the manufacturer’s instructions. The remaining ground tissue was boiled at 100°C for 2.5 h and then neutralized by 5 mol l^–1^ KOH (VWR, United States). The pH of the solution was then adjusted to 7.4 and total glucose (free glucose + glucose obtained from glycogen hydrolysis) was measured using the same kit as before. Glycogen content was evaluated by subtracting free glucose levels. Total lipid content was determined gravimetrically after extraction by dichloromethane/methanol (2:1, v/v), containing 0.01% of butylated hydroxytoluene (BHT) as antioxidant, according to Folch et al. (1957). Total protein content was measured using the Kjeldahl method as for diet analysis.

### Plasma Metabolite and Sex Steroid Analyses

Plasma glucose, triglycerides, cholesterol and free fatty acids (FFA) were analyzed with Glucose RTU (BioMerieux, Marcy-l’Étoile, France), PAP 150 (BioMerieux), FUJIFILM WAKO (Sobioda) and NEFA C (Wako Chemicals GmbH, Neuss, Germany) kits, respectively, according to the recommendations of each manufacturer.

Sex steroids were extracted from total plasma samples as described by McMaster et al. (1992), using 100 μl of serum as starting volume for repeated diethyl ether extraction, and a final assay buffer reconstitution volume buffer of 500 μl. Isolated steroid fractions were used for quantification of 17β-estradiol in female, and testosterone in male serum samples using the Estradiol ELISA kit (Cayman Chemicals, Ann Arbor, MI, United States) and TEST96 ELISA kit (Teco Diagnostics, Anaheim, CA, United States) according to the manufacturers’ instructions. Samples were prediluted to fall into assay sensitivity range based on expected seasonal ranges of circulating sex steroids (Baynes and Scott, 1985; Liley et al., 1986a,b; Sundling et al., 2014). Serum steroid concentrations were calculated based on a regression formula obtained from standard curve absorbance readings fitted by 4 parameters logistic regression with *R*^2^ > 0.99, taking into account additional predilution of samples.

### Oocyte and Sperm Fatty Acid Analysis

Fatty acid profiles of 6 individual spawns per diet and of a pool of sperm per diet were analyzed. Fatty acid methyl esters (FAME) were prepared from lipid extracts by acid-catalyzed transmethylation, using boron trifluoride according to Shantha and Ackman (1990). FAME were then analyzed in a Varian 3900 gas chromatograph equipped with a fused silica DB Wax capillary column (30 m × 0.25 mm internal diameter, film thickness 0.25 μm; JW Alltech, France). Injection volume was 1 μl, using helium as carrier gas (1 ml/min). The temperatures of the injector and the flame ionization detector were 260°C and 250°C, respectively. The thermal gradient was as follows: 100–180°C at 8°C/min, 180–220°C at 4°C/min and a constant temperature of 220°C for 20 min. Fatty acids were identified with reference to a known standard mixture (Sigma, St. Louis, MO, United States) and peaks were integrated using Varian Star Chromatography Software (Star Software, version 5). The results for individual FA were expressed as percentage of total identified FAME.

### Oocyte Density and Diameters Determination

At each sampling, a piece of ovary (∼5–7 g) from 4 sampled females per condition (except for the last sampling for which the 8 spawns per condition were evaluated) was kept in a saline buffer and dissected. The diameter of 600–1000 oocytes per female was measured using the ImageJ^1^ measurement plugin. Plots (Figure 7D) showing the density of oocytes depending on diameters were obtained using R Commander package in R (density option) (v.3.1.0) (Fox, 2005).

### RNA Extraction and RT-qPCR

Total RNA extraction and cDNA synthesis: The analysis of mRNA levels was conducted in the liver, ovary and testis tissue. Samples were homogenized in Trizol reagent (Invitrogen, Carlsbad, CA, United States) with Precellys^®^24 (Bertin Technologies, Montigny-le-Bretonneux, France), and total RNA was then extracted according to the Trizol manufacturer’s instructions. Total RNA (1 μg) was subsequently reverse transcribed to cDNA in duplicate using the SuperScript III RNase H-Reverse Transcriptase kit (Invitrogen) with random primers (Promega, CharbonnieÌres-les-Bains, France).

Quantitative real-time PCR assays: The primers used in quantitative real-time PCR (qPCR) assays as well as gene abbreviations are summarized in Supplementary Table S1. qPCR assays were performed with the Roche Lightcycler 480 system (Roche Diagnostics, Neuilly-sur-Seine, France). The reaction mix was 6 μl per sample, including 2 μl of diluted cDNA template (1:76), 0.12 μl of each primer (10 μmol l-1), 3 μl of Light Cycler 480 SYBR^®^ Green I Master mix and 0.76 μl of DNase/RNase-free water (5 Prime GmbH, Hamburg, Germany). The qPCR protocol was initiated at 95°C for 10 min for the initial denaturation of the cDNA and hot-start Taq-polymerase activation, followed by 45 cycles of a two-step amplification program (15 s at 95°C; 10 s at 60°C). Melting curves were monitored systematically (temperature gradient 0.11°C s^–1^ from 65 to 97°C) at the end of the last amplification cycle to confirm the specificity of the amplification reaction. Each qPCR assay included replicate samples (duplicate of reverse transcription and PCR amplification) and negative controls (reverse-transcriptase and cDNA-template-free samples). The relative quantification of mRNA levels of target genes were normalized to the transcript abundance of reference genes (For liver: RS16: forward 5′-TTTCAGGTGGCGAAACATGC-3′, reverse 5′-GGGGTCTGCCATTCACCTTG-3′; and for ovaries and testis by a geometric mean of mRNA levels of the following genes as previously proposed by Vandesompele et al. (2002): β-actin er: forward 5′-GATGGGCCAGAAAGACAGCTA-3′, reverse 5′-TCGTCCCAGTTGGTGACGAT-3′; ef1α: forward 5′-TCCTCTTGGTCGTTTCGCTG-3′, reverse-5′-ACCCGAGGGA CATCCTGTG-3′, RS16, RL27: forward 5′-CACAACCATGGG CAAGAAGA-3′, reverse 5′-TCAGGGCAGGGTCTCTGAAG-3′, 18s: forward 5′-CGGAGGTTCGAAGACGATCA-3′, reverse 5′-TCGCTAGTTGGCATCGTTTAT-3′) using the E-method on Light Cycler software.

### Enzymatic Activities

Enzymatic activities were measured in frozen samples of livers and gonads. An ice-cold buffer [50 mmol/l TRIS, 5 mmol/l EDTA, 2 mmol/l DTT, protease inhibitor cocktail (Sigma, St. Louis, MO, United States)] was used to grind the samples. For the fatty acid synthase (Fasn) and the glucose-6-phosphate dehydrogenase (G6pd), after homogenization, samples were centrifuged for 20 min at 24,000 g and supernatants were kept for the enzymatic assay. For all the remaining enzymes, once homogenized samples were centrifuged for 10 min at 900 g at 4°C and supernatants were kept for the enzymatic assay. A supplementary centrifugation (20 min at 20,000 g) was applied to measure the pyruvate kinase (Pk) and the phosphofructokinase (Pfk) activities. For phosphoenolpyruvate carboxykinase (Pck) activities and the glucose-6-phosphatase (G6pc), a sonic disruption (Bioruptor, 3 cycles, 30 s On/30 s Off), followed by a second centrifugation (10 min at 900 g at 4°C) was applied and supernatants were kept for the enzymatic assay.

Enzyme activities were measured following the variation of absorbance of nicotinamide adenine dinucleotide phosphate at 340 nm, in a Power Wave X (BioTek Instrument) reader. The reactions were started by the addition of the specific substrate. Water was used as a blank for each sample. The enzymes assayed were: high Km Hexokinase (Gck), as described by Panserat et al. (2000), G6pc from Alegre et al. (1988), Pk and Pck following the protocol of Kirchner et al. (2003), fructose-1,6-bisphosphatase (Fbp) as described by Tranulis et al. (1996), Pfk according to Metón et al. (2004), G6pd was assayed according to the methods of Bautista et al. (1988), Fasn according to the protocol of Chakrabarty and Leveille (1969). Finally, glycogen synthase (GSase) activity was measured as described in Polakof et al. (2008). Total GSase activity, which represents activities of the active and the non-active forms, along with the percentage of the active form (% GSase *a*) were estimated. One unit of enzyme activity was defined as the amount of enzyme that catalyzed the hydrolysis of 1 μmol of substrate per minute. Enzyme activity was expressed per milligram of tissue.

### Statistical Analysis

All of the statistical analyses were performed with the R Software (version 3.5.2). The results are presented as mean ± SD.

The PCA analyses were performed with the packages “FactoMineR” and “missMDA,” as appropriate. A dataset of the variables concerning growth performance, biochemical composition of different tissues and enzymatic activities were used to run the PCA analyses in order to determine the key variables.

Statistical analyses were carried out on the different parameters recorded to test the effects of the different factors: diet, month and the interaction between diet and month. After a verification of the condition of application of ANOVA, two-way ANOVAs were performed on the different variables obtained. When an interaction was found to be significant, means of all treatments were compared by a Tukey’s *post hoc* analysis. Concerning the mRNA levels in ovulated oocytes, the volume of sperm and the concentration of spermatozoids, the effect of the diet was analyzed by a Student’s *T*-test.

## Results

### Results Obtained in Males

#### PCA Analysis

**FIGURE 2:**
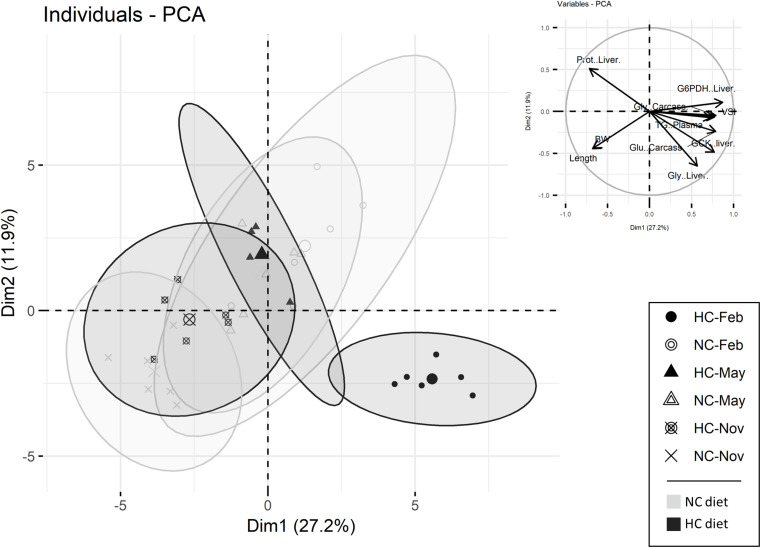
Principal components analysis (PCA) of male data. Individuals are represented either by circles (sampled in February), by triangles (sampled in May) or by a cross (sampled in November). The top 15 variables, which participate in the construction of the PC1 and PC2, are also represented. (NC, no carbohydrate diet; HC, high carbohydrate diet; Feb., February; Nov., November).

A principal components analysis (PCA) was performed in order to discriminate between individuals and to determine which variable were the most informative among the ones measured during the year (growth parameters, biochemical compositions of tissues, plasma metabolites and enzymatic activities) (Figure 2). The first and second principle components (PC1 and PC2) explained 27.2 and 11.9% of the variability, respectively. The two components separated males fed the HC diet sampled in February from the other individuals (Figure 2). PC1 and PC2 were interpreted by inspecting the correlations of the different variables, to reflect mostly modification of carcass and liver biochemical composition and the hepatic metabolism (gluconeogenesis and lipid metabolism). The results obtained are further detailed in the next sections of the results.

#### Growth Parameters

While the body weight and length significantly increased throughout the year (Table 2), these two parameters were also significantly affected by diet; with a lower final body weight/length in November for males fed the HC diet.

**TABLE 2:** Effect of HC diet during spermatogenesis on growth, biochemical composition of tissues and plasma metabolites.

	February		May		November		*p*-value		
	NC	HC	NC	HC	NC	HC	Diet	Month	diet:month
Body weight (g)	607 ± 178	645 ± 172	741 ± 205	663 ± 246	1779 ± 253	1092 ± 322	**1E−04**	**4E−03**	0.571
Lenght (cm)	33.3 ± 2.7	35.5 ± 3.1	36.8 ± 2.9	36.1 ± 4.8	49.8 ± 3.5	42.8 ± 4.0	**6E−03**	**1E−03**	0.335
**Carcass**									
Protein (%DM)	59.5 ± 6.1	54.4 ± 3.9	58.5 ± 5.7	59.1 ± 4.3	62.2 ± 4.3	60.8 ± 7.6	0.514	0.252	0.239
Lipid (%DM)	29.1 ± 7.0	35.4 ± 4.4	32.7 ± 6.2	32.8 ± 4.5	28.2 ± 4.7	30.4 ± 8.9	0.358	0.462	0.268
Glucose (mg⋅g^–1^)	1.08 ± 0.10^b^	1.77 ± 0.37^a^	0.73 ± 0.17^bc^	0.29 ± 0.13^c^	0.44 ± 0.17^c^	0.42 ± 0.30^c^	0.252	**3E−10**	**3E−05**
Glycogen (mg⋅g^–1^)	13.3 ± 5.3^ab^	17.6 ± 6.7^a^	8.9 ± 3.7^bc^	1.2 ± 0.8^d^	1.7 ± 0.9^d^	4.7 ± 0.9^cd^	0.377	**2E−06**	**2E−03**
**Liver**									
HSI (%)	1.3 ± 0.3^bc^	2.1 ± 0.3^a^	1.7 ± 0.6^ab^	1.5 ± 0.4^abc^	1.0 ± 0.1^c^	1.0 ± 0.1^c^	7E−02	**3E−04**	**2E−03**
Protein (%DM)	63.2 ± 2.3^b^	40.9 ± 4.8^c^	64.1 ± 2.9^b^	72.7 ± 5.0^a^	63.3 ± 5.1^b^	70.3 ± 5.0^ab^	**4E−05**	**8E−11**	**4E−09**
Lipid (%DM)	21.2 ± 12.3	23.6 ± 7.2	10.0 ± 0.6	10.0 ± 0.8	10.5 ± 0.9	11.0 ± 1.4	0.868	**7E−05**	0.665
Glucose (mg⋅g^–1^)	58.8 ± 12.8^a^	22.2 ± 6.26^c^	29.3 ± 8.53^bc^	52.8 ± 32.2^ab^	63.8 ± 9.54^a^	59.0 ± 4.84^a^	**0.003**	**2E−05**	**7E−05**
Glycogen (mg⋅g^–1^)	52.2 ± 41.7^b^	316.8 ± 60.7^a^	83.3 ± 40.8^b^	29.8 ± 43.3^b^	154.5 ± 72.1^b^	57.6 ± 66.4^b^	**6E−05**	**5E−06**	**1E−06**
**Digestive Tract**									
VSI (%)	9.5 ± 2.1	11.6 ± 1.6	7.4 ± 0.6	7.2 ± 0.6	6.5 ± 0.9	8.0 ± 2.0	0.061	**4E−05**	0.077
Protein (%DM)	42.8 ± 12.9	23.0 ± 8.7	49.8 ± 7.2	20.6 ± 6.6	50.6 ± 23.0	39.6 ± 17.0	**1E−03**	0.068	0.454
Lipid (%DM)	46.7 ± 17.2	68.7 ± 7.0	44.7 ± 11.0	76.5 ± 7.7	50.2 ± 27.4	58.1 ± 21.2	**5E−03**	0.310	0.521
**Gonads**									
GSI (%)	1.4 ± 0.6	2.9 ± 1.3	1.5 ± 1.1	2.1 ± 1.3	3.0 ± 0.7	3.2 ± 1.7	**0.009**	**0.004**	0.569
Protein (%DM)	91.3 ± 7.1	97.3 ± 4.0	95.3 ± 5.7	97.4 ± 5.6	90.5 ± 9.5	95.6 ± 5.6	0.146	0.629	0.490
Lipid (%DM)	8.2 ± 1.6	7.0 ± 0.7	7.8 ± 0.9	8.2 ± 1.2	6.6 ± 0.6	7.7 ± 1.0	0.128	0.767	0.092
Glucose (mg⋅g^–1^)	0.21 ± 0.07	0.16 ± 0.08	0.19 ± 0.11	0.30 ± 0.03	0.07 ± 0.02	0.14 ± 0.08	0.677	**0.028**	0.405
Glycogen (mg⋅g^–1^)	0.66 ± 0.18	0.58 ± 0.20	0.65 ± 0.12	0.48 ± 0.15	0.30 ± 0.02	0.36 ± 0.10	0.183	**0.006**	0.507
**Plasma Metabolites**									
Glucose (g⋅L^–1^)	1.1 ± 0.2	1.8 ± 0.6	0.8 ± 0.1	1.6 ± 1.5	1.1 ± 0.1	1.1 ± 0.1	**0.007**	0.182	0.060
Triglycerides (g⋅L^–1^)	11.2 ± 5.1^ab^	16.8 ± 6.8^a^	15.2 ± 7.4^a^	1.6 ± 1.2^c^	4.7 ± 1.9^bc^	4.7 ± 0.8^bc^	0.394	**3E−05**	**9E−05**
FFA (mmol⋅L^–1^)	0.4 ± 0.2	0.4 ± 0.1	0.3 ± 0.1	0.4 ± 0.2	0.3 ± 0.1	0.5 ± 0.1	0.495	0.837	0.124
Cholesterol (g⋅L^–1^)	4.1 ± 0.9^a^	3.6 ± 0.7^ab^	3.9 ± 1.0^ab^	2.0 ± 0.6^b^	4.2 ± 1.4^a^	3.6 ± 0.7^ab^	**0.007**	0.292	**0.01**

*Data are presented as means ± SD (n = 8 fish for body weight/length/VSI/HIS/GSI and plasma metabolites, and n = 6 for biochemical composition except in May for males fed the HC diet n = 4) and analyzed by two-way ANOVA (P-values < 0.05 in bold), followed by a post hoc Tukey test in case of significant interaction. In this latter case, mean values not sharing a common lowercase letter are significantly different from each other. HIS, hepato-somatic index; VSI, viscero-somatic index; GSI, gonado-somatic index; FFA, Free Fatty Acids; NC, no carbohydrate diet; HC, high carbohydrate diet; DM, dry matter.*

#### Biochemical Composition of Carcasses and Tissues

An analysis of the biochemical composition of the carcass revealed that protein and lipid content remained stable throughout the year, independent of diet (Table 2). Overall, the content in free glucose and glycogen decreased during the year (Table 2). In February, the glycogen and free glucose contents of males fed the HC diet were significantly higher than those obtained in other fish (Table 2 and Figure 2).

The hepato-somatic index (HSI) as well as the biochemical compositions of the liver were significantly affected over the course of the year, by diet and by the interactions between these two factors. Only the lipid content significantly varied during the year (Table 2). During the experiment, the HSI decreased and was significantly lower in November. This decrease was associated with a decrease in the lipid content (Table 2). In the liver tissue of males fed the NC diet, free glucose was higher in February compared to males fed the HC diet, whereas the opposite was true in May, with the free glucose concentration reaching its maximum observed in November regardless of the diet (Table 2). In February, as revealed in the PCA analysis, males fed the HC diet had a significantly higher HSI correlated with a higher glycogen content but a lower protein content (Table 2 and Figure 2).

The viscero-somatic index (VSI) varied during the year and was significantly higher in February in comparison to the end of the year, irrespective of the diet (Table 2). Both lipid and protein content remained stable over the course of the experiment, but for males overall, those fed the HC diet had a higher lipid and a lower protein content than fish fed the control diet (Table 2).

The gonado-somatic-index (GSI) increased at the end of the year (November) and overall, fish fed the HC diet had a higher GSI than fish fed the NC diet. Nevertheless, the biochemical composition of testes was not affected by the diet but only by the time of the year (Table 2). The lipid and protein contents of testes remained stable, whereas the free glucose and the glycogen contents significantly diminished throughout the year (Table 2).

#### Male Plasma Metabolites

In males, only the plasma triglyceride concentration varied over the course of the year, with an overall decrease in its concentration. In contrast, except for the FFA concentrations, all measured plasma metabolites were affected by diet. Males fed the HC diet had a lower plasma cholesterol concentration during the entire year and a lower triglyceride concentration in May compared to other fish (Table 2). Plasma glucose concentration was significantly higher in fish fed the HC diet compared to the control fish, regardless of the time of the year, and for most of the samples, its value never exceeded 1.8 g⋅L^–1^.

#### Glucose and Lipid Metabolism in Liver and Testes

**FIGURE 3:**
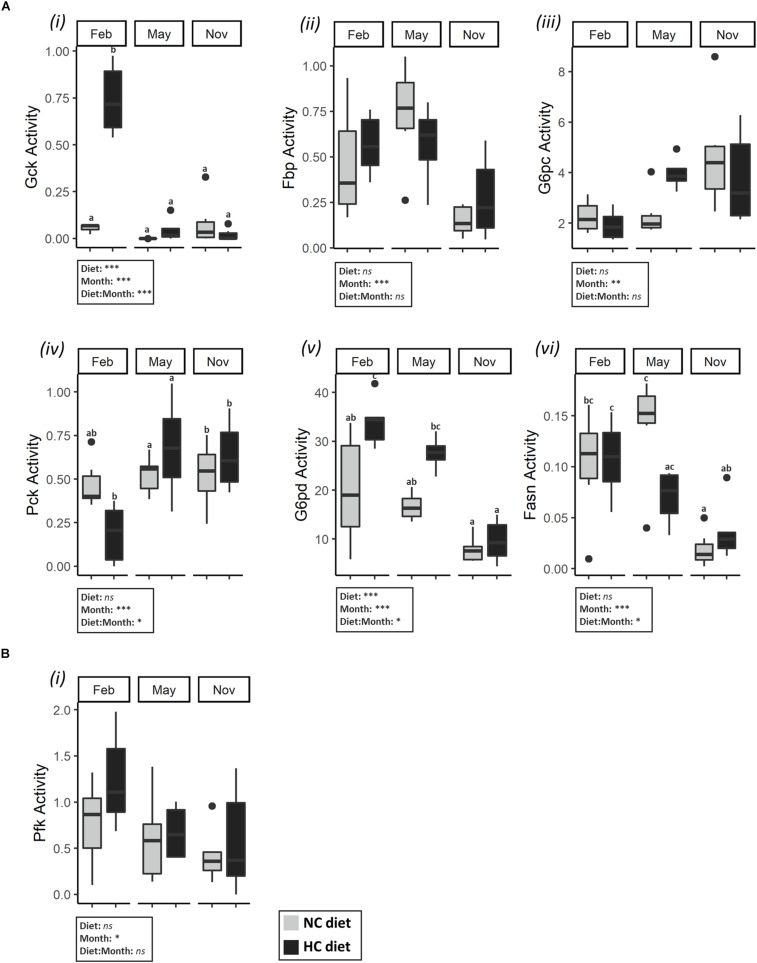
Activities of enzymes related to carbohydrate and lipid metabolism in the liver and gonads of males. Enzymatic activities (expressed in U⋅g^–1^ of tissue) **(A)** (i) Gck, (ii) Fbp, (iii) G6pc, (iv) Pck, (v) G6pd, (v) Fasn, in the liver in February, May, November; and **(B)** (i) Pfk in testes in February, May and November. Data were analyzed using a two-way ANOVA (with *p < 0.5, **p < 0.1, ***p < 0.001), followed by a *post hoc* Tukey test in case of significant interaction (significant differences are indicated with different letters). NC, no carbohydrate diet; HC, high carbohydrate diet; ns, not significant; Feb., February; Nov., November.

In the liver, both carbohydrate and lipid metabolisms were modified during the year and were affected by the HC diet (Figure 3A and Supplementary Tables S2, S3). Concerning glycolysis, all of the mRNA levels measured fluctuated during the year but only the *gcka* and *gckb* mRNA levels were affected by diet (Supplementary Table S3). These results are supported by Gck activity as males fed the HC diet had a significantly higher activity of Gck in the liver in February compared to fish fed the NC diet, as well as for fish sampled at other time points (Figure 3A). Concerning the gluconeogenesis pathway, the activities of Fbp and the G6pc varied significantly during the year, but the observed patterns were different for these two enzymes (Figure 3A). Moreover, the mRNA levels of *pck1* were affected by the interaction between the diet and the time of the year (Supplementary Table S3). This result is supported by Pck activity, and males fed the HC diet tended to have lower gluconeogenesis Pck enzyme activity in February (Figure 3A). Finally, concerning lipid metabolism, in February, fish fed the HC diet had higher *G6pd* mRNA levels and a higher activity of G6pd in liver, compared to fish fed the NC diet (Figure 3A). The activity of Fasn changed throughout the year and significantly decreased from February to November. As described by the PCA analysis, males fed the HC diet in February were differentiated from the other fish with respect to their Gck, Pck and G6pd activities (Figure 2).

In testes, enzyme activities in the tested enzymes were not significantly affected by the month nor by the diet (Supplementary Tables S2, S4), except for the activity of Pfk. Pfk activity decreased over time irrespective of the diet (Figure 3B).

### Results Obtained in Females

#### PCA Analysis

**FIGURE 4:**
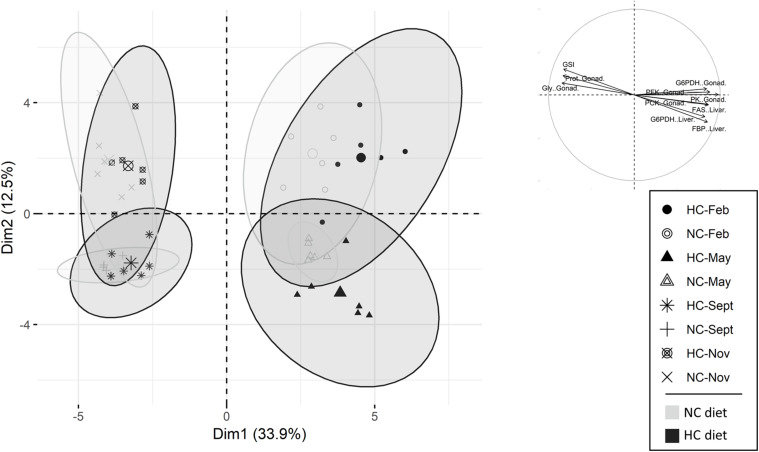
Principal components analysis (PCA) of female data. Individuals are represented either by circles (sampled in February), by triangles (sampled in May), by a plus (sampled in September) or by a cross (sampled in November). The top 15 variables, which participate in the construction of the PC1 and PC2, are also represented. NC, no carbohydrate diet; HC, high carbohydrate diet; Feb., February; Sept., September; Nov., November.

In females, the first and the second principle components, PC1 and PC2 explained 33.9 and 12.7%, respectively, of the variability (Figure 4). The PCA separated fish into four separate clusters based on sampling time. More precisely, PC1 separated the females sampled at the beginning of the trial (February and May) from the ones sampled at the end of the trial (September and November). The PC2 separated, with little overlap, females sampled in February from those sampled in May, and fish sampled in September from fish sampled November (Figure 4). PC1 consisted mostly of variables describing the biochemical composition of the ovaries during the year. Within each cluster obtained by the PCA, fish fed the NC diet were not different from fish fed the HC diet (Figure 4). The results obtained are further detailed in the following sections of the results.

#### Growth Parameters of Females

Female body weight and length significantly increased during the year (Table 3) and these two parameters were never significantly affected by the HC diet.

**TABLE 3:** Effect of HC diet during oogenesis on growth, biochemical composition of tissues and plasma metabolites.

	February		May		September		November		*p*-value		
	NC	HC	NC	HC	NC	HC	NC	HC	Diet	Month	d:m
Body weight (g)	1841 ± 279	1512 ± 248	2466 ± 359	2418 ± 664	3247 ± 489	3168 ± 477	2628 ± 247	2765 ± 404	0.511	**6.00E−10**	0.603
Lenght (cm)	49 ± 1.5	47.2 ± 1.5	51.1 ± 2.1	50.6 ± 3.5	56.2 ± 3.6	56.3 ± 2.4	56 ± 1.4	54.8 ± 3.5	0.256	**1.00E−09**	0.835
**Carcass**											
Protein (%DM)	56.5 ± 4.8	57.9 ± 5.7	57.3 ± 2.9	53.7 ± 5.4	57.8 ± 1.6	58.9 ± 3.1	58.9 ± 4.8	59.2 ± 1.8	0.865	0.171	0.404
Lipid (%DM)	35.4 ± 5.4	32.9 ± 5.9	35.8 ± 3	39.6 ± 6.4	36.4 ± 1.9	34.4 ± 3.3	31.8 ± 5	32.3 ± 2.9	0.961	**0.028**	0.294
Glucose (mg.g^–1^)	0.38 ± 0.27^b^	0.75 ± 0.16^a^	0.75 ± 0.09^a^	0.70 ± 0.07^a^	0.22 ± 0.05^b^	0.43 ± 0.14^b^	0.29 ± 0.09^b^	0.29 ± 0.09^b^	**2E−03**	**2E−09**	**2E−03**
Glycogen (mg⋅g^–1^)	2.1 ± 1	3.0 ± 1.5	3.7 ± 1.2	6.5 ± 6.2	2.5 ± 0.3	1.5 ± 1.3	3.1 ± 1.0	6.0 ± 0.9	**0.046**	**7E−03**	0.172
**Liver**											
HSI (%)	1.0 ± 0.1^c^	1.5 ± 0.5^bc^	1.4 ± 0.1^c^	2.0 ± 0.3^ab^	2.1 ± 0.1^a^	2.3 ± 0.2^a^	1.1 ± 0.2^c^	1.0 ± 0.2^c^	**1E−04**	**2E−10**	**0.024**
Protein (%DM)	69.9 ± 4.4^ab^	45.4 ± 9.3^d^	62.4 ± 2.8^bc^	54.3 ± 7.0^cd^	77.0 ± 0.9^a^	72.1 ± 2.4^a^	76.2 ± 4.3^a^	76.1 ± 2.6^a^	**6E−08**	**1E−13**	**2E−06**
Lipid (%DM)	20.7 ± 10.6	20.9 ± 11.7	14.5 ± 2.7	13.2 ± 2.4	13 ± 0.5	12 ± 0.5	10.8 ± 0.4	11.2 ± 0.5	0.814	**7E−04**	0.977
Glucose (mg⋅g^–1^)	51.9 ± 13.9^a^	49.7 ± 16.4^a^	22.1 ± 6.99^b^	12.0 ± 7.04^bc^	2.61 ± 0.32^c^	6.96 ± 2.13^bc^	25.2 ± 8.11^b^	46.3 ± 10.8^a^	0.060	**2E−13**	**3E−03**
Glycogen (mg⋅g^–1^)	51.4 ± 63.5^c^	406.7 ± 181.2^a^	63 ± 38.5^c^	224 ± 65.9^b^	20.9 ± 11.8^c^	230.1 ± 45.9^b^	64.0 ± 73.4^bc^	18.8 ± 18.7^c^	**1E−09**	**2E−06**	**1E−05**
**Digestive Tract**											
VSI (%)	8.7 ± 1.0^d^	8.4 ± 1.7^d^	9.8 ± 1.1^d^	9.6 ± 1.0^d^	13.1 ± 1.3^c^	13.4 ± 1.7^c^	21.8 ± 2.3^a^	17.9 ± 2.7^b^	**0.043**	**2E−16**	**0.015**
Protein (%DM)	21.7 ± 4.5^b^	21.6 ± 5.6^b^	22.9 ± 4.7^b^	20.5 ± 6.5^b^	22.6 ± 6^b^	14.9 ± 3.3^b^	42.1 ± 20.9^a^	16.9 ± 7.3^b^	**2E−03**	**0.037**	**6E−03**
Lipid (%DM)	73.1 ± 6.9^ab^	76.2 ± 9.2^ab^	74.4 ± 5.2^ab^	75.3 ± 6.6^ab^	78 ± 9.3^ab^	86.5 ± 3.2^a^	61.2 ± 19.3^b^	83.8 ± 7.7^a^	**3E−03**	0.085	**0.036**
**Gonads**											
GSI (%)	0.3 ± 0.0^d^	0.3 ± 0.1^d^	0.4 ± 0.1^d^	0.5 ± 0.2^d^	6.7 ± 1.3^c^	5.6 ± 1.7^c^	17.5 ± 2.2^a^	13.3 ± 2.1^b^	**1E−03**	**2E−16**	**6E−04**
Protein (%DM)	60.9 ± 3.4	62 ± 4.3	50.3 ± 1.2	53.2 ± 4	67.9 ± 1.2	68.3 ± 1	71.2 ± 1	71.4 ± 1.8	0.134	**2E−16**	0.594
Lipid (%DM)	16.8 ± 2.3	15.6 ± 2.4	22.2 ± 0.7	21.1 ± 2.7	18.5 ± 1	16.9 ± 0.9	15.8 ± 0.4	15.1 ± 0.7	**0.021**	**4E−11**	0.954
Glucose (mg⋅g^–1^)	0.59 ± 0.13^b^	1.35 ± 0.30^a^	0.41 ± 0.08^bcd^	0.46 ± 0.06^bc^	0.17 ± 0.03^e^	0.19 ± 0.07^de^	0.28 ± 0.03^cde^	0.31 ± 0.04^cde^	**9E−07**	**2E−16**	**5E−09**
Glycogen (mg⋅g^–1^)	0.52 ± 0.07	1.28 ± 0.67	0.50 ± 0.22	1.32 ± 0.36	1.54 ± 0.29	1.90 ± 0.27	2.40 ± 0.45	2.73 ± 0.69	**4E−05**	**2E−12**	0.349
**Plasma Metabolites**											
Glucose (g⋅L^–1^)	1.3 ± 0.52	1.61 ± 0.9	0.6 ± 0.1	0.9 ± 0.2	0.7 ± 0.1	1.2 ± 0.3	1.1 ± 0.2	1.0 ± 0.2	**0.030**	**2E−05**	0.301
Triglycerides (g⋅L^–1^)	8.9 ± 3.5	8.7 ± 3.1	13.4 ± 5.7	17.3 ± 7.1	10.0 ± 1.8	11.3 ± 2.7	4.3 ± 1.3	5.1 ± 0.8	0.151	**7E−09**	0.523
FFA (mmol⋅L^–1^)	0.47 ± 0.19	0.34 ± 0.18	0.29 ± 0.07	0.34 ± 0.10	0.27 ± 0.05	0.20 ± 0.06	0.24 ± 0.06	0.28 ± 0.20	0.368	**0.002**	0.186
Cholesterol (g⋅L^–1^)	6.3 ± 1.2	5.8 ± 1.3	5.0 ± 0.9	3.9 ± 1.0	3.6 ± 0.7	4.1 ± 0.8	4.1 ± 0.7	3.8 ± 0.5	0.131	**5E−09**	0.103

*Data are presented as means ± SD (n = 8 fish for body weight/length/VSI/HIS/GSI and plasma metabolites, and n = 6 for biochemical composition) and analyzed by two-way ANOVA (P-values < 0.05 in bold), followed by a post hoc Tukey test in case of significant interaction. In this latter case, mean values not sharing a common lowercase letter are significantly different from each other. HIS, hepato-somatic index; VSI, viscero-somatic index; GSI, gonado-somatic index; FFA, Free Fatty Acids; NC, no carbohydrate diet; HC, high carbohydrate diet DM, dry matter.*

#### Biochemical Composition of the Carcass and Tissues

The biochemical composition of the different tissues analyzed was highly modified with respect to the time of year, and moderately by diet (Table 3). While protein content of carcasses remained stable, lipid, free glucose and glycogen contents varied over the year (Table 3). The lipid content of the female carcass only increased in May compared to other sampling times irrespective of the dietary treatment (Table 3). The free glucose concentration decreased over the year with a higher concentration in females fed the HC diet in February, compared to those fed the NC diet. The glycogen content fluctuated seasonally, exhibiting its highest concentration in May. Overall, the glycogen content was significantly higher in the carcasses of females fed the HC diet compared to those fed the NC diet (Table 3).

HSI increased through the year, but decreased significantly in November. Females fed the HC diet had a higher HSI in May compared to fish fed the NC diet (Table 3). Protein content increased during the year but was significantly lower in fish fed the HC diet in February compared to fish fed the NC diet. Conversely, the lipid and glycogen contents decreased throughout the year. The glycogen content was significantly higher in fish fed the HC diet from February to September compared to those fed the NC diet (Table 3). The free glucose content decreased from February to September and increased in November, but in a higher extant in fish fed the HC diet (Table 3).

The VSI significantly increased from May to September, regardless of the diet (Table 3). Concerning the protein content, it remained stable until September and then significantly increased in November in females fed the NC diet. Finally, fish fed the HC diet displayed higher lipid content in their digestive tract in November compared to fish fed the NC diet (Table 3).

During the year, ovaries experienced a drastic shift in their biochemical compositions between May and September (Table 3 and Figure 4). The GSI significantly increased over the year, along with a significant increase in the protein and glycogen contents. The lipid content in ovaries increased in May compared to other months and then slightly decreased at the end of the year. Overall, fish fed the HC diet had a lower GSI in November, but an overall higher glycogen content than fish fed the NC diet. The free glucose content decreased during the year, and was significantly higher in females fed the HC diet in February in comparison to females fed the NC diet.

#### Plasma Metabolites in Females

All the tested plasma metabolites varied during the year. While, the plasma glucose, FFA and cholesterol concentrations significantly decreased, the triglycerides concentration increased in May and then decreased at the end of the trial (Table 3). Similar to what was observed for males, overall, plasma glucose concentration was significantly higher in fish fed the HC compared to the control fish but its value never exceeded 1.6 g⋅L^–1^.

#### Metabolism in the Liver and Ovaries of Females

**FIGURE 5:**
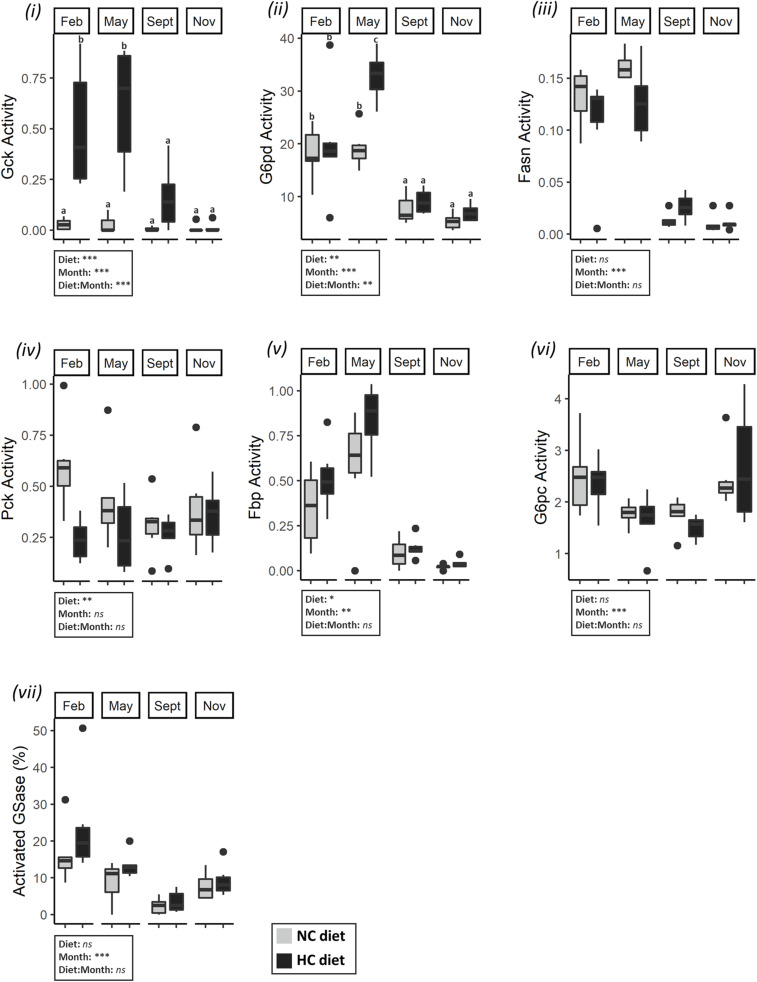
Activities of enzymes related to carbohydrate and lipid metabolism in the liver of females. Enzymatic activities (expressed in U.g^–1^ of tissue) of (i) Gck, (ii) G6pd, (iii) Fasn, (iv) Pck, (v) Fbp, (vi) G6pc, and (vii) GSase in the liver in February, May, September, November. Data were analyzed using a two-way ANOVA (with *p < 0.5, **p < 0.1, ***p < 0.001), followed by a *post hoc* Tukey test in case of significant interaction (significant difference are indicated different letters). NC, no carbohydrate diet; HC, high carbohydrate diet; ns, not significant; Feb., February; Sep., September; Nov., November.

**FIGURE 6:**
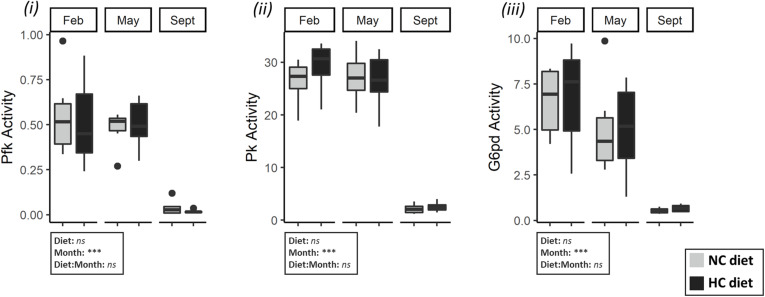
Activities of enzymes related to carbohydrate and lipid metabolism gonads of females. Enzymatic activities (expressed in U.g^–1^ of tissue) of (i) Pfk (ii) Pk (iii) G6pd (iv) Pck in ovaries in February, May and September. Data were analyzed using a two-way ANOVA (with ***p < 0.001), followed by a *post hoc* Tukey test in case of significant interaction (significant difference are indicated different letters). NC, no carbohydrate diet; HC, high carbohydrate diet; ns, not significant; Feb., February; Sep., September; Nov., November.

A consistent pattern was identified for activities of various enzymes involved in carbohydrate and lipid metabolisms in liver and gonads, including the activities of the glycolytic enzymes in the ovaries, the lipogenic enzymes G6pd and Fasn in both tissues, the gluconeogenic enzymes Pck in the gonad and Fbp in the liver and the activity of GSase in liver. All of these enzymes exhibited a high activity in February and May, followed by a sharp decrease at the end of the trial (Figures 5, 6). The activities were negatively correlated with the protein and glycogen content of ovaries as described by the first component of the PCA (Figure 4), and help to differentiate between fish sampled at the beginning of the study with those sampled at the end of the year. G6pc in the liver was also affected by the time of the year, but a lower G6pc activity was shown in May and September in comparison to the other periods of the year (Figure 5). Hepatic activities of Pk, Pfk, Pck, and total GSase were the only ones that remained stable throughout the year (Supplementary Table S5).

Dietary treatment also affected hepatic activities of Gck, G6pd, Pck, and Fbp activities as follows: females fed the HC diet had a significantly higher Gck activity in February and May compared to females fed the NC diet (Figures 4, 5). These females also had a higher *g6pd* mRNA level and a higher G6pd activity in May (Supplementary Table S6 and Figure 4, 5). Finally, results concerning the gluconeogenesis were highly variable. While females fed the HC diet had an overall higher Fbp activity, they had a lower Pck activity. In ovaries, diet only affected the mRNA levels of *fbp1a* and *fbp1b* (Supplementary Table S7) but did not affect enzymatic activity (Supplementary Tables S5).

### Gametogenesis and Reproductive Performance

Surprisingly, males fed the HC diet began to display a mature fish phenotype as early as the end of April by developing a lethal *Saprolegnia* infection and by being milted (47% affected against 0% for males fed the NC diet or for females). Thus, remaining males were individually tagged and transferred in the “NC” tank in May (Figure 1) and survived until November to be used later for reproduction (no sampling was performed in September for males).

#### Spermatogenesis

Steroidogenesis (*fshr, lhcgr* and *star*) related genes (Sambroni et al., 2013) as well as molecular markers of different spermatogenetic stages (Bellaiche et al., 2014) (*nanos2, dazl, piwi2, and plzfb*), were monitored (Supplementary Table S8). These results showed that *dazl* mRNA level decreased from February to May and *fshr* as well as *nanos2* mRNA levels were higher in males fed the HC diet in May compared to males fed the NC diet.

**FIGURE 7:**
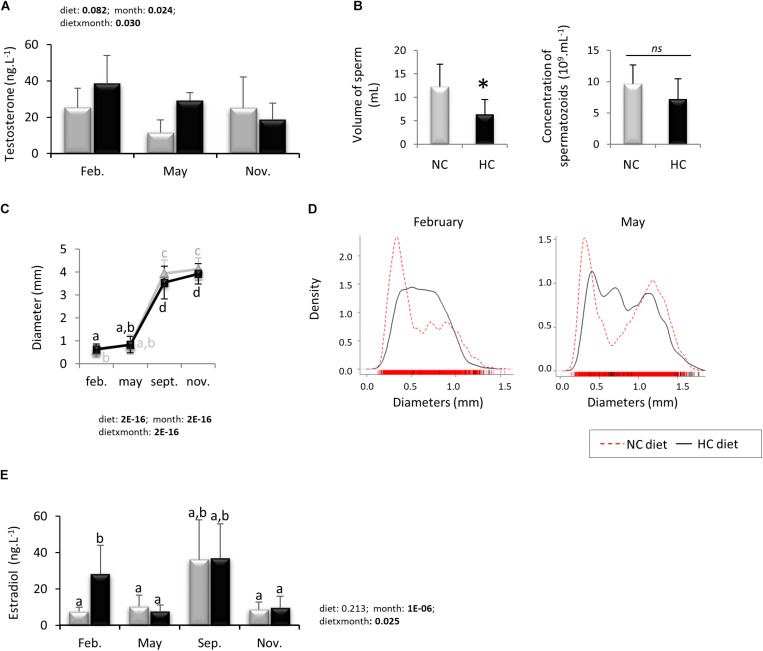
Reproductive parameters of broodstock during the whole reproductive cycle. Plasmatic steroids concentrations were measure in males (**A**, testosterone) and in females (**E**, estradiol) fed with both diets. The volume of sperm collected and its concentration in cells **(B)** were measured in November year 2 in males fed the NC (*n* = 8) or the HC (*n* = 4) diet. The diameter of oocytes per g of tissue during the whole reproductive cycle **(C)** and their density repartition in February and May **(D)** were also monitored. When applicable, data are presented as mean ± SD. Data presented in **(B)** were analyzed by a Student’s *T*-test whereas other data **(A,C,E)** were analyzed using a Two-ways ANOVA followed by a *post hoc* Tukey test in case of significant interaction (significant difference are indicated by a star and different letters, respectively). NC, no carbohydrate diet; HC, high carbohydrate diet; ns, not significant; Feb., February; Sep., September; Nov., November.

Male plasma testosterone concentration was measured for both dietary groups during the year. Time of the year, diet and the interaction of both factors significantly affected circulating testosterone concentrations (Figure 7A). However, while testosterone concentration tended to be higher in trout fed the HC diet in May compared to the males fed the NC diet, Tukey’s *post hoc* test did not resolve specific differences between groups.

In November, only 4 of the 8 remaining males produced milt and while the volume of the collected milt was significantly lower for males fed the NC diet compared to HC diet, the spermatozoa concentration was identical between both groups (Figure 7B).

#### Oogenesis

At each sampling point during the year, 4 ovaries per condition (or ovulated oocytes from 8 females at spawning period) were dissected to estimate the diameters of oocytes. The oocyte diameter increased over the course of the year and was significantly higher in HC fed females in February compared to the NC fed females, while these differences reversed in September and November (Figure 7C). We then estimated the density of oocytes as a function of their diameters (presented as the mean of all females in Figure 7D, or per female in Supplementary Figure S2). These results showed that in February most of the oocytes from NC fed females had diameters between 0 and 0.5 mm, while oocytes from HC fed females displayed a larger distribution between 0 and 1.25. In May, oocytes from NC fed females distributed into two main peaks (one at 0.25 mm and the other at 1.25 mm) whereas again oocytes from HC fed females had a larger distribution from 0 to 1.5 mm.

The estradiol (E2) plasma concentration was measured during the year (Figure 7E) and our results showed that E2 was higher in females fed the HC diet in February compared to females fed the NC diet.

mRNA levels of genes related to yolk precursor protein production (*vtg, er*α*1*, and *er*α*2*) in liver (Supplementary Table S9A) and molecular markers of different oogenetic stages (*cyp19a1a, cxcl14, aqp4*, and *apoc1*) (Bobe et al., 2009) in ovaries were analyzed (Supplementary Table S9B). Except for *fshr* and *apoc1*, mRNA levels of these genes were significantly increased in September but none were affected by diet.

#### Gametes Characteristics

Because the volume of stripped sperm collected in November was limiting, and in particular for males fed the HC diet, we chose to keep this sperm for cross-fertilizations and thus did few analyses on stripped sperm (all data collected in November for biochemical composition and gene expression were performed on whole milted testis). Fatty acid profiles of sperm from males fed the NC and HC diets were very similar, however, these data for information purposes only as they were obtained from pools of sperm and no replications were done (Supplementary Table S10).

For females, as previously described, no significant differences at the molecular level (Supplementary Table S9) were detected between ovulated oocytes from those fed the NC diet and those fed the HC diet. No significant effects of the diet were detected on the protein, lipid and glycogen contents of ovulated oocytes (Table 3, see gonads in November). We also analyzed the fatty acid profile of oocytes collected from six individual spawns per dietary condition (Table 4). These results showed that oocytes from females fed the HC diet had significantly higher 16:1, 20:3 n-6 and 20:4 n-6 but lower 18:4 n-3, 20:4 n-3 and 20:5 n-3 contents than oocytes from females fed the NC diet.

**TABLE 4:** FA profiles in ovulated oocytes from spawns (% total FA).

	NC	HC	*p-*value
**Saturated**			
14:0	3.84 ± 0.30	4.10 ± 0.56	0.200
15:0	0.48 ± 0.05	0.52 ± 0.07	0.200
16:0	24.43 ± 0.63	23.47 ± 1.22	0.200
18:0	4.56 ± 0.13	4.24 ± 0.38	0.109
*Total*	33.31 ± 0.85	32.33 ± 1.95	0.522
**MUFA**			
16:1	10.11 ± 0.65	11.56 ± 1.12	**0.025**
18:1	28.12 ± 1.06	27.73 ± 1.40	0.522
20:1	1.06 ± 0.19	1.07 ± 0.15	0.423
*Total*	39.29 ± 1.14	40.36 ± 2.19	0.423
**PUFA n-6**			
18:2 n-6	3.82 ± 0.79	3.53 ± 0.46	0.522
20:2 n-6	0.39 ± 0.06	0.46 ± 0.10	0.200
20:3 n-6	0.25 ± 0.10	0.52 ± 0.16	**0.006**
20:4 n-6	1.12 ± 0.09	1.54 ± 0.20	**0.004**
*Total*	5.58 ± 0.89	6.05 ± 0.68	0.150
**PUFA n-3**			
18:3 n-3	1.10 ± 0.29	0.80 ± 0.15	0.055
18:4 n-3	0.28 ± 0.06	0 ± 0	**0.002**
20:4 n-3	0.39 ± 0.05	0.12 ± 0.15	**0.010**
20:5 n-3	4.78 ± 0.35	3.88 ± 0.38	**0.004**
22:5 n-3	0.76 ± 0.22	0.77 ± 0.34	1
22:6 n-3	11.94 ± 2.09	12.88 ± 2.68	0.631
*Total*	19.24 ± 2.07	18.45 ± 3.11	0.522
*LC-PUFA n-3*	17.87 ± 2.26	17.65 ± 3.05	0.631
**Sat/PUFA**	1.34 ± 0.13	1.32 ± 0.24	0.749
**n3/n6**	3.45 ± 0.71	3.05 ± 0.40	0.150

*Data are presented as means ± SD (n = 6 spawns) and analyzed by a Student T-test (P-values < 0.05 in bold), followed by a post hoc Tukey test in case of significant interaction. In this latter case, mean values not sharing a common lowercase letter are significantly different from each other. MUFA, monosaturated fatty acids; PUFA, polyunsaturated fatty acids; LC-PUFA, long chain polyunsaturated fatty acids; Sat, saturated; n-6, omega 3; n-6, omega 6; NC, no carbohydrate diet; HC, high carbohydrate diet.*

#### Reproductive Performance

During the spawning period in November, we performed cross-fertilizations between 2 NC females, 3 HC females and 4 males from each treatment (Supplementary Figure S1). The survival at eyed stage and at hatching reached 89.4 and 86.3%, respectively (Table 5 and Supplementary Table S11). There were no significant differences between crossing concerning the survival of embryos at eyed stage nor the survival of live fish at hatching (Table 5). We also evaluated malformations at hatching and showed that, irrespective of the diet fed by males, alevins from females fed the HC diet displayed significantly fewer malformations (1%) than those fed the HC diet (3%) (Table 5).

**TABLE 5:** Reproductive performance of broodstock fed either the NC or the HC diet.

Female diet	Male diet	Survival at eyed stage (%)	Survival at hatching (%)	Malformation (%)
NC	NC	90.2 ± 6.5	85.9 ± 8.9	3.3 ± 1.8^a^
NC	HC	88.2 ± 7.3	83.5 ± 10.4	3.1 ± 1.5^a^
HC	NC	87.7 ± 6.4	85.6 ± 7.0	1.1 ± 0.9^b^
HC	HC	91.4 ± 2.5	90.3 ± 2.7	1.0 ± 1.2^b^
*p*-value		0.492	0.604	**6.7E−04**

*Data are presented as means ± SD and analyzed by a Kruskal–Wallis rank sum test (P-values < 0.05 in bold). NC, no carbohydrate; HC, High carbohydrates.*

## Discussion

The present study aimed to evaluate the impact of a low-protein high-carbohydrate diet administered to both mature male and female rainbow trout. While the consequences of diets containing a high amount of highly digestible carbohydrates have been widely studied on juvenile fish (Polakof et al., 2012; Kamalam et al., 2017), their effects on broodstock metabolism have received little attention.

### Female Broodstock Were Able to Eat the Low-Protein High Carbohydrate for an Entire Year

In our experiment, we were able to confirm that broodstock were capable of consuming the HC diet, thanks to different parameters. First, *gck* mRNA levels and Gck activity in the liver are known to be induced by the ingestion of glucose in trout (Panserat et al., 2000) and Gck activity reflects the ingestion and metabolization of dietary carbohydrates. In the light of data obtained for Gck, we were able to confirm that broodstock consumed the HC diet throughout the entire year for females and in February for males. Secondly, broodstock fed the HC diet displayed an increase in glycemia, which remained within a normal glycemic range. Finally, the changes observed in the biochemical composition of tissues regarding free glucose and the storage of glycogen revealed that different tissues have incorporated and stored glucose when fish were fed the HC diet. A higher proportion of free glucose (+63% for males in February) and glycogen (+49% in females regardless of the period) were retrieved in carcasses (predominantly muscle tissue) of fish fed the HC diet. Similar results were found for the liver from both males and females, which also stored some carbohydrates provided by the HC diet in the form of glycogen.

Altogether, we demonstrated that female broodstock were able to consume a low-protein high-carbohydrate diet over an entire reproductive cycle. For males, the results are promising as they were also able to consume a low-protein high-carbohydrate diet for at least the early stages of the reproductive cycle. However, conclusions on the ability of male broodstock to consume such a diet over an entire reproductive cycle cannot be drawn because in the present experiment, males most likely stopped consuming the HC diet in May, due to a *Saprolegnia* sp. infection.

### The Low-Protein High-Carbohydrate Diet Induced Precocious Maturation

Unexpectedly, some males fed the HC diet were mature as early as the end of April. This early maturation unfortunately triggered a *Saprolegnia* sp. infection, a parasite known to mainly infect mature salmonids and preferentially mature males (Richards and Pickering, 1978) due to a change in the structure of their epidermis, which render fish more susceptible to infection (Pickering and Willoughby, 1982). We thus hypothesized that the HC diet induced precocious male sexual maturation. This hypothesis was confirmed by higher transcript abundance of *fshr* mRNA along with the higher concentration of plasma testosterone in males fed the HC diet in comparison to males fed the NC diet in May. Interestingly, although phenotypically less visible, a precocious maturation appeared to affect females as well, given that a higher concentration of plasma E2 was detected in females fed the HC diet in February compared to those fed the NC diet. Finally, some oocytes had already begun vitellogenesis in February in females fed the HC diet, but not the NC diet, further supporting this conclusion.

The high proportion of carbohydrates in the HC diet could be the cause of the early sexual maturation; and here we propose different mechanisms in support of this hypothesis.

Firstly, dietary carbohydrates could cause precocious maturation in females by modulating the hormonal control of this process, as it has already been shown in female pigs (Li et al., 2016). In this latter study, the dietary carbohydrates increased the level of secreted insulin, which led to an over-secretion of GnRH from the hypothalamus, increasing LH and FSH levels and thereby the production of E2. In fish, the insulin-like growth factor-I (IGF-I) is known to be highly affected by glucose and in turn has a more effective impact on glucose metabolism than insulin (Enes et al., 2011). Furthermore, IGF-1 is known to be involved in sexual maturation in both male and female fishes (Reinecke, 2010); either by a direct endocrine action on both male and females gonads as shown by the stimulated effect of IGF-1 on spermatogenesis and oocytes diameters in eels (Nader et al., 1999; Lokman et al., 2007), or by interaction with LH and FSH (Wood et al., 2005).

Secondly, the timing of sexual maturity is also modulated by a variety of factors including energy balance and the onset of puberty in fish. While sexual maturity occurs when fish reach a certain combination of age and size, the accumulation of energy reserves needed for reproduction (Taranger et al., 2010; Juntti and Fernald, 2016) also plays a role. In the present study, the HC diet also led to an augmentation of glycogen storage in carcass and liver in males in February and for the whole year in females. Although the lipid reserves seem to be the most crucial determinant concerning the timing of salmonid maturation (Taranger et al., 2010), a study demonstrated that early-maturing amago salmon have higher glycogen storage in comparison to late-maturing individuals in spring (Silverstein et al., 1997). Even though our two experimental diets were isoenergetic, dietary carbohydrates could have induced precocious maturation, either directly by a modification in hormonal response, and/or by modifying fish energy reserves. However, the onset of gonadal maturation involves a multitude of factors and it could be not excluded that other mechanisms were involved and/or interact with the ones proposed here.

However, it is worth noting that a high variability in stage of maturation was also present in our population. For instance, while *nanos2* mRNA is known to be more abundantly expressed in immature trout testes (Bellaiche et al., 2014), our data revealed that it was also more abundantly expressed in May for males fed the HC diet. Similar results were found in females as demonstrated by the wide variability of densities of oocytes in function of their diameters (Supplementary Figure S2). As it was previously pointed out by Soengas et al. (1993a, b), intraspecific variation exists in the timing of the reproductive cycle in both sexes in trout (Billard, 1992; Schultz et al., 2003). In this experiment, fish were sampled at different points during the year to cover the reproductive cycle and results are discussed in relation to these points in time rather than a specific stage of maturation. Indeed, in aquaculture farms, broodstock are usually raised as groups of individuals of the same age, and in this sense, our aim here was to describe what happened in such a group of broodstock during a whole year without considering the individual timing of reproductive cycle.

### Gamete Characteristics and Reproductive Performance Were Not Affected by the Low-Protein High Carbohydrate Diet

Although the timing of sexual maturation was affected in fish fed the HC diet, reproductive performance (survival at eyed stage, at hatching and rate of malformations) were high and in the same range as the ones previously obtained in the same facility (Wischhusen et al., 2019). More importantly, the diet did not negatively impair survival rates. These results are in accordance with previous data obtained in which female broodstock were fed a high carbohydrate diet (Luquet and Watanabe, 1986; Washburn et al., 1990).

For females, while the diameter of oocytes was slightly affected by the HC diet (larger oocytes at the beginning of the year and smaller ones at the end), their biochemical composition was not affected (except for FA profiles) but the differences observed were not biologically significant. Females were thus able to produce oocytes of high quality. Overall, our findings indicate for the first time that feeding female broodstock a low-protein high-carbohydrate diet throughout an entire reproductive cycle led to good gamete quality and reproductive performance. However, as only two females fed the control diet and three females fed the HC diet were used for the cross-fertilizations, these results will have to be confirmed in a future experiment.

For males, the present study was only a preliminary analysis as only pools of sperm were analyzed. However, no differences were detected in the FA composition of sperm. While these results are promising, they need to be confirmed in a future experiment, as we cannot exclude that we selected males that were unaffected by the *Saprolegnia* infection.

### The Low-Protein High-Carbohydrate Diet Did Not Affect Female Growth Performance

Although the HC diet induced early maturation, it did not affect growth performance in females fed the HC diet throughout the year, nor did it affect males in February.

While the impact of a low-protein high-carbohydrate diet on broodstock has never been investigated on males, a previous study performed on females, with a diet containing white flour as carbohydrate source suggested an inhibitory effect on the growth rate of females (Washburn et al., 1990). This difference in relation to our current study could stem from the lower digestibility of their experimental diet (i.e., white flour versus gelatinized starch that we used here) (Washburn et al., 1990). For males, the growth rate of fish fed the HC diet began to decrease after May. This growth reduction was mostly linked to the *Saprolegnia* sp. infection that occurred in spring. Overall, no conclusions on the effect of the HC diet could thus be drawn from our results obtained in May and in November.

The poor ability of high trophic level fish to use dietary carbohydrates is often correlated to the inability of fish to clear glucose from the bloodstream, triggering a persistent postprandial hyperglycemia. This phenotype is typically observed in juveniles fed a diet containing more than 20% of digestible carbohydrates (Polakof et al., 2012; Kamalam et al., 2017). In the present study, broodstock were never hyperglycemic during the feeding trial, confirming the improved ability of broodstock to use dietary carbohydrates, as previously suggested (Luquet and Watanabe, 1986; Washburn et al., 1990; Barciela et al., 1993; Soengas et al., 1993b). Different tissues (liver, carcass and ovaries) took up and stored a portion of the glucose from the bloodstream.

Overall, we demonstrated here for the first time that a low-protein high-carbohydrate diet did not impair broodstock growth performance probably because the fish were able to efficiently metabolize and/or store digestible dietary carbohydrates in different tissues.

### Carbohydrate Metabolism Changed Drastically During Gametogenesis in Both Sexes and Irrespective of Diet

The present study also allowed us to identify glucose metabolism changes during gametogenesis in broostock fed a low-protein high-carbohydrate diet. During gametogenesis, there is a strong trade-off between growth, maintenance, and reproduction, generating important metabolic change, as is certainly the case for carbohydrate metabolism (Barciela et al., 1993; Soengas et al., 1993a, b). In liver, gametogenesis is typically characterized by an augmentation of glycolysis, and a decrease of gluconeogenesis (Barciela et al., 1993; Soengas et al., 1993a, b). In gonads, there is a strong increase in glucose levels, which come from the bloodstream and from an endogenous production in gonads in the first stages (Barciela et al., 1993; Soengas et al., 1993a, b). This glucose appears to be used as substrate for glycogen synthesis and for the production of reducing power (Barciela et al., 1993; Soengas et al., 1993a, b). Then, in both liver and gonads, a decrease in enzymatic activities involved in the glycolysis and the pentose phosphate pathways (PPP) indicates a reduction in the need for glucose as an energy source during the latter stages (Barciela et al., 1993; Soengas et al., 1993a, b).

Unlike results described in previous studies (Barciela et al., 1993; Soengas et al., 1993a, b), modulations of carbohydrate metabolism during the first stages of gametogenesis were not identified in our study, neither in the liver nor in gonads. Some effects could be masked by the high variability observed in our data, probably resulting from the presence of various sexual maturation stages in our populations. Nevertheless, our observations confirmed a general pattern, with an initial high-energy demand in both liver and gonads at the onset of gametogenesis, as shown by high activities of enzymes involved in the glycolysis and the PPP in these two tissues and a low gluconeogenesis in liver. This stage was then followed by a drop in different enzymatic activities in both females and males (Gck and G6pd in liver; Pfk, Pk, and G6pd activities in ovaries; Pfk in testis). This high-energy demand of broodstock during gametogenesis and their ability to modulate their glucose metabolism is one of the main hypotheses to explain this striking difference between juveniles and broodstock in response to dietary carbohydrates. Therefore, we hypothesized that during the first stages of gametogenesis, diet-derived carbohydrates could have been used in the different pathways stimulated during gametogenesis such as the glycolysis to produce energy and the PPP to produce reducing power needed for lipid and protein synthesis.

### The Low-Protein High-Carbohydrate Diet Induces Hepatic PPP and Glycogenesis

The HC diet strongly induced the first step of glycolysis in liver by enhancing Gck activity, in the same way as what is typically observed in juveniles (Panserat et al., 2000), confirming that both males and females metabolized the produced glucose in the liver. It could however be noted that Gck activity was not induced in May in males fed the HC diet and this pattern obtained during spring is comparable to the one obtained in November when fish were fasted. In May, fish fed the HC diet probably stopped eating, due to the *Saprolegnia* infection. These results could explain the reduction in growth and the diminished effect of the HC diet observed in May in males. Surprisingly, for both males and females, the following steps of glycolysis were not significantly affected by this diet.

In February and May for males fed the HC diet, and in May for females, the glucose-6-phosphate (G6P) produced by Gck appears to be used in the PPP. A similar effect was previously shown in juveniles fed a high carbohydrate diet (Dias et al., 1998). The reducing power produced by this pathway could be used to synthesize lipids, but because the HC diet contained a lower proportion of protein, a macronutrient known to induce lipogenesis in trout liver (Dai et al., 2015) and in European sea bass (Viegas et al., 2016), fish fed the HC diet did not display a higher Fasn activity. For perivisceral tissues, it was previously demonstrated that salmonids have a low ability to convert glucose into cellular lipids for storage (Bou et al., 2016). Nevertheless, the augmentation of the lipid content in these tissues suggests that a small proportion of the produced glucose may also be used as substrate for lipogenesis. The fate of NADPH, other than for lipogenesis, as well as ribose 5-phosphate, both of which could have been produced by PPP to a higher degree when fish were fed the HC diet, require further study.

In addition to the enhancement of the PPP, an important part of the dietary carbohydrates was stored as glycogen in the liver, causing a higher HSI for both sexes. This result is in line with previous results obtained in salmonids (Hemre and Storebakken, 2000; Hemre et al., 2002). In accordance with this phenotype, females fed the HC diet tended to have an augmented proportion of the active form of GSase in the liver throughout gametogenesis (Supplementary Table S4). Surprisingly, this was not the case for males (Supplementary Table S1), and different sampling times after the first meals should be performed to have a general view of the activation of this pathway during the short-term.

Interestingly, the precocious maturation observed in females may have also contributed to their increased ability to use dietary carbohydrates *via* the PPP and the glycogenesis as E2 is known to lower the concentration of plasmatic somatostatin in rainbow trout (Holloway et al., 2000) stimulating glycogenolysis (Eilertson et al., 1991) and reducing PPP (Eilertson et al., 1991; Polakof et al., 2012).

Finally, one of the hypotheses proposed to explain the reduced ability of GI species to use dietary carbohydrates is their inability to inhibit the endogenous production of glucose in the liver when fed a highly digestible carbohydrate diet (Marandel et al., 2015). More specifically, a previous study on juveniles detected two *g6pc* ohnologs (*g6pcb2a* and *g6pcb2b*) which have an atypical up-regulation of their expression with such a diet (Marandel et al., 2015). Until now, no molecular data were available regarding the capacity of broodstock to repress this pathway when fed low-protein high carbohydrate diets. Thus, our findings showed for the first time that while some of the broodstock responses regarding this pathway were similar to the ones observed in juvenile GI species (Marandel et al., 2015), some of them contrasted with these latter ones. Interestingly, the expression of the two ohnologs, *g6pcb2a* and *g6pcb2b*, were not significantly enhanced by the HC diet in the male liver. In females, Pck activity was significantly reduced in response to high dietary carbohydrates, in contrast to the pattern observed in juveniles (Marandel et al., 2015). Finally, both females and males fed the HC diet did not show any stimulation of the activities of the enzymes involved in the hepatic gluconeogenesis. These patterns observed here are remarkably similar to the ones observed in omnivorous fish (Panserat et al., 2002), which are known to have a higher glucose tolerance (Legate et al., 2001). These findings suggest that a better regulation of the gluconeogenesis pathway in the liver could be one of the reasons why broodstock have a greater ability to use dietary carbohydrates.

### The Low-Protein High-Carbohydrate Diet Affected Male and Female Gonads Differently

While hepatic responses (composition, metabolites, and metabolism) in male and female were very similar, the response of the two sexes greatly differed in the gonads. While testis seemed little affected by the diet, as no modification of their biochemical compositions, nor alteration of glucose metabolism was detected, ovaries were highly impacted by the HC diet. Females fed the HC diet had a higher content of free glucose in the ovary in February and an overall higher storage of glycogen, which could be further used as a source of energy at the end of gametogenesis (Mommsen and Walsh, 1988). However, the glucose metabolism in the ovaries was not affected by the diet. Unlike testis, ovaries thus seem to contribute to the females’ ability to use dietary carbohydrates, taking up and storing part of the carbohydrate intake.

Surprisingly, broodstock of a GI species were able to modulate their carbohydrate metabolism with the HC diet. Of particular interest, dietary lipids are known to strongly interact with carbohydrate metabolism (Kamalam et al., 2017). Fish nutritional requirements vary during a fish’s lifespan, and broodstock, in comparison to juveniles, only require a small proportion of lipids in their diet (in the present experiment, the experimental diets contains only 9% lipids). Previous studies in juveniles demonstrated that a diminution of dietary lipid inclusion typically led to both the enhancement of the activities of the glycolytic enzymes Hk and Gck, but also G6pd involved in the PPP, combined with the diminution of the G6pc activity, involved in gluconeogenesis in both liver and muscle (Figueiredo-Silva et al., 2012). The modulation of the glucose metabolism observed in the present study, which may be involved in the higher ability of broodstock to use dietary carbohydrates compared to juveniles, could thus also stem from the low dietary lipid inclusion. Further experiments should be carried out to better understand to what extent the interaction between macronutrients (and more particularly glucose and lipids) is involved in broodstock responses to high levels of dietary carbohydrates in liver. Moreover, as in this experiment the first sampling occurred almost 2 months after the first feeding, it will be essential to determine how rapidly broostock are able to implement their response.

## Conclusion

Here, we demonstrate that trout broodstock, known as a GI fish species, were able to consume a low-protein high-carbohydrate diet over an entire reproductive cycle for females and for at least the early part of the cycle for males. Of particular interest, broodstock were never hyperglycemic while fed such a diet. This result is in strong contrast with previous results obtained in juveniles of GI species. Molecular analyses reveal that the liver appears to be the main tissue that responds to the low-protein high-carbohydrate diet. The main responses observed were an augmentation of the PPP, the glycogenesis and a potentially better ability to regulate gluconeogenesis. Finally, although both males and females fed the HC diet displayed precocious maturation, the reproductive performance was unaffected by the low-protein high carbohydrate diet.

These results are highly promising and suggest that dietary carbohydrates can at least partially replace proteins in broodstock aquafeed. For males, further studies are however needed to adapt the content of carbohydrates in their diet to feed them over an entire reproductive cycle. Further studies, which investigate the potential subsequent effect on progeny, are also required, as it is now well known that broodstock nutritional history could affect progeny phenotype and metabolism in the long-term (Lazzarotto et al., 2016). This will be of particular importance as carbohydrates have been shown to act as epigenetic modulators in rainbow trout (Marandel et al., 2016).

## Data Availability Statement

All datasets generated for this study are included in the article/Supplementary Material.

## Ethics Statement

The animal study was reviewed and approved by French National Consultative Ethics Committee.

## Author Contributions

PM and NT took care of fish the whole year and organized sampling. HH and AS performed biochemical analysis. JL and EP-J extracted RNA and performed RT-qPCR. TC performed enzymatic activities analyses. JM measured steroid plasma concentrations. JB brought his expertise into salmonid reproduction and molecular markers of maturations. CB designed formula of the diets. GC brought her expertise into lipids and FA profiles during reproduction and analyzed related results. SP brought his expertise into glucose metabolism in trout, was involved in the experimental plan design and contributed to the correction of the manuscript. LM designed and managed the study. LM and TC performed statistical analysis and wrote the manuscript. All authors read and approved the final version of the manuscript.

## Conflict of Interest

The authors declare that the research was conducted in the absence of any commercial or financial relationships that could be construed as a potential conflict of interest.

## Footnotes

1 http://rsbweb.nih.gov/ij/index.html
