# Supplementary material for: Exploring the Impact of a Low-Protein High-Carbohydrate Diet in Mature Broodstock of a Glucose-Intolerant Teleost, the Rainbow Trout
Source: Front Physiol. 2020 May 15;11:303. doi: 10.3389/fphys.2020.00303 (PMC7243711; doi:10.3389/fphys.2020.00303)
Supplement: Supplementary file 2 [file Table_2.DOCX]

|  | **February** | | | | | | |  | **May** | | | | | | |  | **November** | | | | | | | | *p-value* | |  |
| --- | --- | --- | --- | --- | --- | --- | --- | --- | --- | --- | --- | --- | --- | --- | --- | --- | --- | --- | --- | --- | --- | --- | --- | --- | --- | --- | --- |
|  | **NC** |  |  |  | **HC** |  |  |  | **NC** |  |  |  | **HC** |  |  |  | **NC** |  |  |  | **HC** |  |  |  | *Diet* | *Month* | *Diet:Month* |
| **Liver** |  |  |  |  |  |  |  |  |  |  |  |  |  |  |  |  |  |  |  |  |  |  |  |  |  |  |  |
| Gck | 0.06 | ± | 0.02^a^ |  | 0.74 | ± | 0.19^b^ |  | 0.00 | ± | 0.00^a^ |  | 0.05 | ± | 0.07^a^ |  | 0.08 | ± | 0.13^a^ |  | 0.02 | ± | 0.03^a^ |  | **5.96E-08** | **7.83E-10** | **8.69E-10** |
| Pfk | 6.00 | ± | 1.98 |  | 5.42 | ± | 1.16 |  | 4.15 | ± | 1.80 |  | 6.23 | ± | 1.86 |  | 4.10 | ± | 1.09 |  | 4.90 | ± | 1.51 |  | 1.92E-01 | 1.71E-01 | 1.63E-01 |
| Pk | 2.21 | ± | 0.36 |  | 2.04 | ± | 1.01 |  | 2.31 | ± | 0.67 |  | 2.27 | ± | 0.86 |  | 2.01 | ± | 0.87 |  | 2.20 | ± | 0.63 |  | 9.79E-01 | 8.11E-01 | 8.39E-01 |
| Pck | 0.47 | ± | 0.14^ab^ |  | 0.19 | ± | 0.16^b^ |  | 0.53 | ± | 0.11^a^ |  | 0.68 | ± | 0.31^a^ |  | 0.53 | ± | 0.18^b^ |  | 0.63 | ± | 0.19^b^ |  | 6.25E-01 | **2.33E-03** | **1.44E-02** |
| Fbp | 0.46 | ± | 0.31 |  | 0.57 | ± | 0.16 |  | 0.74 | ± | 0.28 |  | 0.57 | ± | 0.24 |  | 0.15 | ± | 0.08 |  | 0.28 | ± | 0.22 |  | 7.46E-01 | **9.17E-05** | 2.56E-01 |
| G6pc | 2.26 | ± | 0.61 |  | 1.92 | ± | 0.56 |  | 2.32 | ± | 0.87 |  | 3.98 | ± | 0.70 |  | 4.61 | ± | 2.03 |  | 3.76 | ± | 1.79 |  | 9.66E-01 | **1.34E-03** | 8.30E-02 |
| G6pdh | 20.06 | ± | 11.09^ab^ |  | 33.86 | ± | 4.85^c^ |  | 16.64 | ± | 2.70^ab^ |  | 27.55 | ± | 3.79^bc^ |  | 7.75 | ± | 2.46^a^ |  | 9.62 | ± | 4.21^a^ |  | **9.15E-05** | **3.00E-08** | **3.66E-02** |
| Fasn | 0.10 | ± | 0.05^bc^ |  | 0.11 | ± | 0.04^c^ |  | 0.14 | ± | 0.05^c^ |  | 0.07 | ± | 0.03^ac^ |  | 0.02 | ± | 0.02^a^ |  | 0.04 | ± | 0.03^ab^ |  | 3.50E-01 | **5.53E-06** | **2.74E-02** |
| GSase (Total) | 1.91 | ± | 0.82^ab^ |  | 1.49 | ± | 0.54^ab^ |  | 1.04 | ± | 0.44^ab^ |  | 2.35 | ± | 1.36^ab^ |  | 1.10 | ± | 0.49^ab^ |  | 2.33 | ± | 0.57^b^ |  | **7.57E-03** | 9.70E-01 | **8.92E-03** |
| % GSase *a* | 26.90 | ± | 15.42 |  | 17.58 | ± | 3.99 |  | 20.25 | ± | 11.32 |  | 26.76 | ± | 16.97 |  | 7.83 | ± | 6.17 |  | 7.49 | ± | 5.52 |  | **1.42E-03** | 6.64E-01 | 2.25E-01 |
|  |  |  |  |  |  |  |  |  |  |  |  |  |  |  |  |  |  |  |  |  |  |  |  |  |  |  |  |
| **Testes** |  |  |  |  |  |  |  |  |  |  |  |  |  |  |  |  |  |  |  |  |  |  |  |  |  |  |  |
| Pfk | 0.77 | ± | 0.45 |  | 1.24 | ± | 0.51 |  | 0.62 | ± | 0.50 |  | 0.68 | ± | 0.31 |  | 0.41 | ± | 0.27 |  | 0.58 | ± | 0.56 |  | 1.02E-01 | **2.62E-02** | 5.47E-01 |
| Pk | 6.43 | ± | 3.52 |  | 4.41 | ± | 1.31 |  | 3.98 | ± | 1.31 |  | 4.51 | ± | 1.19 |  | 3.71 | ± | 1.00 |  | 4.30 | ± | 1.90 |  | 6.63E-01 | 1.63E-01 | 1.98E-01 |
| G6pd | 1.69 | ± | 1.41 |  | 1.64 | ± | 0.51 |  | 1.23 | ± | 0.55 |  | 1.12 | ± | 0.54 |  | 1.09 | ± | 0.31 |  | 1.46 | ± | 0.61 |  | 6.60E-01 | 2.89E-01 | 7.05E-01 |
| Pck | 0.42 | ± | 0.24 |  | 0.21 | ± | 0.07 |  | 0.28 | ± | 0.17 |  | 0.32 | ± | 0.10 |  | 0.24 | ± | 0.26 |  | 0.27 | ± | 0.12 |  | 4.12E-01 | 7.14E-01 | 2.23E-01 |

**Supplementary Table 2.** Activities of enzymes involved in carbohydrate and lipid metabolism in livers and gonads of males. Data are presented as means ± SD (n=6 fish except for males fed the HC diet in May n=4) and analysed by two-ways ANOVA followed by a post-hoc Tukey test in case of significant interaction. In this latter case, mean values not sharing a common lowercase letter are significantly different from each other. NC: no carbohydrate diet. HC: high carbohydrate diet. GSase (Total) represents the activity of both the active and the non-active form of the glycogen synthase and % GSase a represent the percentage of the active form. Gck, Fbp, G6pc, Fasn and GSase were also analysed in gonads but activities could have not detected.
