# Supplementary material for: Exploring the Impact of a Low-Protein High-Carbohydrate Diet in Mature Broodstock of a Glucose-Intolerant Teleost, the Rainbow Trout
Source: Front Physiol. 2020 May 15;11:303. doi: 10.3389/fphys.2020.00303 (PMC7243711; doi:10.3389/fphys.2020.00303)
Supplement: Supplementary file 3 [file Table_3.DOCX]

|  | **February** | | | | | | |  | **May** | | | | | | |  | **November** | | | | | | |  | *p*-value | | |
| --- | --- | --- | --- | --- | --- | --- | --- | --- | --- | --- | --- | --- | --- | --- | --- | --- | --- | --- | --- | --- | --- | --- | --- | --- | --- | --- | --- |
|  | **NC** | | |  | **HC** | | |  | **NC** | | |  | **HC** | | |  | **NC** | | |  | **HC** | | |  | diet | month | diet:month |
| *gcka* | 0.36 | ± | 0.34^a^ |  | 2.97 | ± | 1.72^b^ |  | 0.04 | ± | 0.06^c^ |  | 0.03 | ± | 0.06 ^c^ |  | 2.12 | ± | 1.41^a,b^ |  | 0.39 | ± | 0.41^a,b^ |  | 0.378 | **3E-03** | **8E-05** |
| *gckb* | 0.19 | ± | 0.29^a^ |  | 5.22 | ± | 2.77^b^ |  | 0.07 | ± | 0.12^a^ |  | 0.03 | ± | 0.07^a^ |  | 0.10 | ± | 0.14^a^ |  | 0.08 | ± | 0.17^a^ |  | **2E-04** | **3^E^-05** | **1E-05** |
| *pfkla* | 1.88 | ± | 0.90 |  | 1.49 | ± | 0.54 |  | 0.68 | ± | 0.31 |  | 1.08 | ± | 0.33 |  | 0.95 | ± | 0.67 |  | 0.61 | ± | 0.28 |  | 0.499 | **1E-03** | 0.270 |
| *pfklb* | 1.79 | ± | 0.68 |  | 1.25 | ± | 0.48 |  | 0.58 | ± | 0.24 |  | 0.92 | ± | 0.29 |  | 0.62 | ± | 0.25 |  | 0.74 | ± | 0.42 |  | 0.752 | **1E-04** | 0.078 |
| *pklr* | 1.75 | ± | 0.63 |  | 0.97 | ± | 0.41 |  | 0.74 | ± | 0.18 |  | 0.46 | ± | 0.32 |  | 0.75 | ± | 0.12 |  | 0.86 | ± | 0.77 |  | 0.070 | **2E-03** | 0.108 |
| *pck1* | 3.73 | ± | 6.71 |  | 0.42 | ± | 0.28 |  | 0.84 | ± | 1.48 |  | 14.17 | ± | 16.13 |  | 0.32 | ± | 0.27 |  | 0.59 | ± | 0.63 |  | 0.217 | 0.177 | **0.027** |
| *pck2* | 1.70 | ± | 2.23 |  | 0.86 | ± | 0.73 |  | 1.10 | ± | 1.08 |  | 1.70 | ± | 1.57 |  | 0.95 | ± | 0.69 |  | 2.36 | ± | 1.26 |  | 0.458 | 0.888 | 0.182 |
| *fbp1a* | 1.74 | ± | 0.70 |  | 1.12 | ± | 0.25 |  | 0.98 | ± | 1.06 |  | 7.59 | ± | 10.23 |  | 1.15 | ± | 0.71 |  | 1.81 | ± | 2.19 |  | 0.168 | 0.268 | 0.106 |
| *fbp1b1* | 1.96 | ± | 0.53 |  | 1.43 | ± | 0.92 |  | 1.30 | ± | 0.68 |  | 2.31 | ± | 1.15 |  | 0.73 | ± | 0.25 |  | 1.47 | ± | 0.97 |  | 0.196 | 0.086 | 0.070 |
| *fbp1b2* | 1.25 | ± | 0.80 |  | 1.95 | ± | 1.35 |  | 1.26 | ± | 1.62 |  | 2.38 | ± | 1.67 |  | 0.58 | ± | 0.42 |  | 1.87 | ± | 1.59 |  | **0.033** | 0.559 | 0.858 |
| *g6pca* | 1.89 | ± | 0.99^a^ |  | 1.50 | ± | 0.65^a^ |  | 0.47 | ± | 0.30^b^ |  | 1.85 | ± | 0.37^a^ |  | 1.55 | ± | 0.45^a^ |  | 1.37 | ± | 0.56^a,b^ |  | 0.390 | 0.098 | **9E-03** |
| *g6pcb1b* | 2.30 | ± | 2.35 |  | 1.59 | ± | 1.10 |  | 0.92 | ± | 0.99 |  | 2.87 | ± | 3.20 |  | 0.86 | ± | 0.44 |  | 0.62 | ± | 0.32 |  | 0.702 | 0.198 | 0.193 |
| *g6pcb2a* | 2.06 | ± | 2.09 |  | 2.56 | ± | 0.97 |  | 1.28 | ± | 0.59 |  | 0.44 | ± | 0.59 |  | 0.32 | ± | 0.18 |  | 0.21 | ± | 0.16 |  | 0.778 | **5E-04** | 0.391 |
| *g6pcb2b* | 2.04 | ± | 2.20^a,b^ |  | 7.85 | ± | 6.19^a^ |  | 2.03 | ± | 1.57^a,b^ |  | 1.35 | ± | 1.68^a,b^ |  | 1.26 | ± | 0.57^b^ |  | 0.16 | ± | 0.29^b^ |  | 0.207 | **8E-03** | **0.020** |
| *glut1ba* | 1.74 | ± | 0.90 |  | 1.19 | ± | 0.43 |  | 0.70 | ± | 0.31 |  | 3.04 | ± | 4.56 |  | 1.09 | ± | 0.27 |  | 1.47 | ± | 1.19 |  | 0.328 | 0.821 | 0.192 |
| *glut1bb* | 1.77 | ± | 0.55 |  | 1.09 | ± | 0.20 |  | 0.83 | ± | 0.30 |  | 2.60 | ± | 2.56 |  | 1.19 | ± | 0.25 |  | 1.34 | ± | 1.29 |  | 0.410 | 0.754 | 0.055 |
| *glut2a* | 1.53 | ± | 0.69 |  | 1.26 | ± | 0.48 |  | 0.75 | ± | 0.21 |  | 1.32 | ± | 0.33 |  | 1.41 | ± | 0.29 |  | 1.27 | ± | 0.59 |  | 0.910 | 0.148 | 0.136 |
| *glut2b* | 1.82 | ± | 1.05 |  | 0.94 | ± | 0.31 |  | 0.78 | ± | 0.42 |  | 1.07 | ± | 0.13 |  | 1.48 | ± | 0.35 |  | 1.26 | ± | 0.63 |  | 0.168 | 0.127 | 0.111 |
| *g6pdh* | 1.53 | ± | 1.67 |  | 2.19 | ± | 1.13 |  | 1.41 | ± | 0.99 |  | 2.79 | ± | 1.91 |  | 0.44 | ± | 0.24 |  | 0.27 | ± | 0.14 |  | 0.186 | **5E-03** | 0.356 |

**Supplementary Table 3.** mRNA levels of glucose metabolism related genes in male livers. Data are presented as means ± SD (n=6 fish except from male fed the HC diet in May n=4) and analysed by two-ways ANOVA followed by a post-hoc Tukey test in case of significant interaction. In this latter case, mean values not sharing a common lowercase letter are significantly different from each other. NC: no carbohydrate diet, HC: high carbohydrate diet. Abbreviations of genes are clarified in Supplementary Table 1. g6pcb1a, glut1aa and glut1ab were also analysed but not detected by RT-q-PCR
