# Supplementary material for: Exploring the Impact of a Low-Protein High-Carbohydrate Diet in Mature Broodstock of a Glucose-Intolerant Teleost, the Rainbow Trout
Source: Front Physiol. 2020 May 15;11:303. doi: 10.3389/fphys.2020.00303 (PMC7243711; doi:10.3389/fphys.2020.00303)
Supplement: Supplementary file 4 [file Table_4.DOCX]

|  | February | | | | | | |  | May | | | | | | |  | November | | | | | | |  | *p*-value | | |
| --- | --- | --- | --- | --- | --- | --- | --- | --- | --- | --- | --- | --- | --- | --- | --- | --- | --- | --- | --- | --- | --- | --- | --- | --- | --- | --- | --- |
|  | NC | | |  | HC | | |  | NC | | |  | HC | | |  | NC | | |  | HC | | |  | diet | month | diet:month |
| *pfkla* | 0.7 | ± | 0.5 |  | 0.7 | ± | 0.6 |  | 0.7 | ± | 0.5 |  | 2 | ± | 1.0 |  | 0.8 | ± | 0.5 |  | 1.3 | ± | 0.6 |  | **0.014** | 0.146 | 0.071 |
| *pfklb* | 0.9 | ± | 0.5 |  | 0.6 | ± | 0.4 |  | 0.9 | ± | 0.8 |  | 2 | ± | 0.8 |  | 1.1 | ± | 0.4 |  | 1.3 | ± | 0.6 |  | 0.257 | 0.067 | **0.042** |
| *pklr* | 0.3 | ± | 0.3 |  | 0.3 | ± | 0.1 |  | 0.4 | ± | 0.5 |  | 0.9 | ± | 0.4 |  | 0.9 | ± | 0.4 |  | 1.3 | ± | 0.6 |  | 0.089 | **4E-04** | 0.306 |
| *pck2* | 1 | ± | 1.3 |  | 0.5 | ± | 0.9 |  | 1 | ± | 0.8 |  | 2.2 | ± | 1.1 |  | 1.2 | ± | 1.0 |  | 0.8 | ± | 0.7 |  | 0.799 | 0.356 | 0.124 |
| *fbp1a* | 0.8 | ± | 0.3^a,b^ |  | 0.6 | ± | 0.1^a^ |  | 0.5 | ± | 0.3 ^a^ |  | 1 | ± | 0.3 ^a,b^ |  | 1 | ± | 0.3 ^a,b^ |  | 0.9 | ± | 0.2^b^ |  | 0.659 | **0.020** | **0.029** |
| *fbp1b1* | 0.5 | ± | 0.4^a,b^ |  | 0.2 | ± | 0.1 ^a^ |  | 0.7 | ± | 0.4 ^a,b^ |  | 2.2 | ± | 2.0 ^b^ |  | 1.8 | ± | 0.7 ^b^ |  | 1.4 | ± | 0.5 ^b^ |  | 0.568 | **0.001** | **0.013** |
| *fbp1b2* | 0.4 | ± | 0.5 |  | 0.5 | ± | 0.2 |  | 1.2 | ± | 0.5 |  | 1.5 | ± | 0.7 |  | 0.6 | ± | 0.6 |  | 1.2 | ± | 0.5 |  | 0.074 | **0.001** | 0.541 |
| *glut1aa* | 0.8 | ± | 0.5 ^a^ |  | 0.1 | ± | 0.1 ^b^ |  | 0.5 | ± | 0.8 ^a,b^ |  | 1.8 | ± | 1.6 ^a,b^ |  | 2 | ± | 1.0 ^a^ |  | 1 | ± | 0.8 ^a,b^ |  | 0.237 | **0.010** | **0.018** |
| *glut1ab* | 0.6 | ± | 0.6 |  | 0.3 | ± | 0.2 |  | 0.7 | ± | 0.4 |  | 0.9 | ± | 0.5 |  | 1 | ± | 0.6 |  | 0.6 | ± | 0.2 |  | 0.130 | 0.115 | 0.437 |
| *glut1ba* | 0.9 | ± | 0.2 |  | 1.1 | ± | 0.2 |  | 0.7 | ± | 0.2 |  | 1 | ± | 0.3 |  | 0.7 | ± | 0.2 |  | 0.7 | ± | 0.2 |  | 0.058 | **0.030** | 0.448 |
| *glut1bb* | 0.7 | ± | 0.2 |  | 0.6 | ± | 0.2 |  | 1.2 | ± | 0.8 |  | 1.3 | ± | 0.6 |  | 0.9 | ± | 0.3 |  | 0.8 | ± | 0.2 |  | 0.788 | **0.019** | 0.829 |
| *glut2b* | 0.2 | ± | 0.2^a^ |  | 0.2 | ± | 0.3 ^a^ |  | 4 | ± | 1.7^b^ |  | 0.6 | ± | 0.2 ^a^ |  | 1.1 | ± | 0.7 ^a^ |  | 0.7 | ± | 0.7 ^a^ |  | **0.002** | **3E-06** | **0.001** |
| *glut4a* | 0.6 | ± | 0.2 |  | 0.4 | ± | 0.2 |  | 1.4 | ± | 0.8 |  | 1.3 | ± | 0.4 |  | 1 | ± | 0.2 |  | 0.8 | ± | 0.2 |  | 0.231 | **0.001** | 0.991 |
| *glut4b* | 1.2 | ± | 0.6 |  | 1.2 | ± | 0.6 |  | 0.6 | ± | 0.4 |  | 2.1 | ± | 2.6 |  | 1.1 | ± | 0.5 |  | 1.1 | ± | 0.5 |  | 0.241 | 0.945 | 0.128 |
| *glut8* | 0.9 | ± | 0.4 |  | 0.8 | ± | 0.2 |  | 1.1 | ± | 0.9 |  | 1.1 | ± | 0.2 |  | 1 | ± | 0.2 |  | 1 | ± | 0.3 |  | 0.780 | 0.456 | 0.851 |
| *g6pdh* | 0.8 | ± | 0.3 |  | 0.7 | ± | 0.1 |  | 1.3 | ± | 0.8 |  | 1.3 | ± | 0.8 |  | 1.1 | ± | 0.5 |  | 0.8 | ± | 0.3 |  | **0.002** | **0.009** | 0.318 |

**Supplementary Table 4**. mRNA levels of glucose metabolism related genes in male testes. Data are presented as means ± SD (n=6 fish except from male fed the HC diet in May n=4) and analysed by two-ways ANOVA followed by a post-hoc Tukey test in case of significant interaction. In this latter case, mean values not sharing a common lowercase letter are significantly different from each other. NC: no carbohydrate diet, HC: high carbohydrate diet. Abbreviations of genes are clarified in Supplementary Table 1. gcka, gckb, pck1, g6pc paralogs, glut2a glut3, were also analysed but not detected by RT-q-PCR.
