# Supplementary material for: Exploring the Impact of a Low-Protein High-Carbohydrate Diet in Mature Broodstock of a Glucose-Intolerant Teleost, the Rainbow Trout
Source: Front Physiol. 2020 May 15;11:303. doi: 10.3389/fphys.2020.00303 (PMC7243711; doi:10.3389/fphys.2020.00303)
Supplement: Supplementary file 5 [file Table_5.DOCX]

|  | **February** | | | | | | |  | **May** | | | | | | |  | | **September** | | | | | | |  | **November** | | | | | | |  | *p-value* | | |
| --- | --- | --- | --- | --- | --- | --- | --- | --- | --- | --- | --- | --- | --- | --- | --- | --- | --- | --- | --- | --- | --- | --- | --- | --- | --- | --- | --- | --- | --- | --- | --- | --- | --- | --- | --- | --- |
|  | **NC** |  |  |  | **HC** |  |  |  | **NC** |  |  |  | **HC** |  |  |  | **NC** | |  |  |  | **HC** |  |  |  | **NC** |  |  |  | **HC** |  |  |  | *Diet* | *Month* | *Diet:Month* |
| **Liver** |  |  |  |  |  |  |  |  |  |  |  |  |  |  |  |  |  | |  |  |  |  |  |  |  |  |  |  |  |  |  |  |  |  |  |  |
| Gck | 0.03 | ± | 0.03^a^ |  | 0.50 | ± | 0.30^b^ |  | 0.03 | ± | 0.04^a^ |  | 0.61 | ± | 0.30^b^ |  | 0.01 | | ± | 0.01^a^ |  | 0.16 | ± | 0.16^a^ |  | 0.01 | ± | 0.02^a^ |  | 0.01 | ± | 0.02^a^ |  | **3.27E-07** | **5.72E-05** | **4.89E-04** |
| Pfk | 5.99 | ± | 1.06 |  | 3.65 | ± | 2.29 |  | 6.49 | ± | 1.10 |  | 5.64 | ± | 1.50 |  | 5.38 | | ± | 1.42 |  | 5.46 | ± | 3.18 |  | 5.05 | ± | 1.21 |  | 6.05 | ± | 4.13 |  | 4.20E-01 | 6.05E-01 | 3.21E-01 |
| Pk | 2.49 | ± | 0.55 |  | 1.45 | ± | 0.83 |  | 2.12 | ± | 1.22 |  | 1.55 | ± | 0.33 |  | 1.01 | | ± | 0.28 |  | 1.91 | ± | 1.92 |  | 2.00 | ± | 1.14 |  | 2.11 | ± | 1.23 |  | 6.34E-01 | 5.43E-01 | 1.52E-01 |
| G6pd | 18.16 | ± | 5.04^b^ |  | 19.98 | ± | 10.57^b^ |  | 19.15 | ± | 3.67^b^ | | 32.86 | ± | 4.61^c^ |  | 7.64 | | ± | 2.77^a^ |  | 9.08 | ± | 2.24^a^ |  | 5.34 | ± | 1.52^a^ |  | 6.95 | ± | 1.69^a^ |  | **2.01E-03** | **1.01E-12** | **7.21E-03** |
| Fasn | 0.13 | ± | 0.03 |  | 0.11 | ± | 0.05 |  | 0.16 | ± | 0.01 |  | 0.13 | ± | 0.03 |  | 0.01 | | ± | 0.01 |  | 0.03 | ± | 0.01 |  | 0.01 | ± | 0.01 |  | 0.01 | ± | 0.01 |  | 1.21E-01 | **2.00E-16** | 8.56E-02 |
| Pck | 0.60 | ± | 0.22 |  | 0.24 | ± | 0.10 |  | 0.43 | ± | 0.23 |  | 0.27 | ± | 0.18 |  | 0.31 | | ± | 0.15 |  | 0.27 | ± | 0.09 |  | 0.39 | ± | 0.22 |  | 0.36 | ± | 0.14 |  | **4.75E-03** | 3.30E-01 | 8.93E-02 |
| Fbp | 0.35 | ± | 0.21 |  | 0.52 | ± | 0.18 |  | 0.70 | ± | 0.14 |  | 0.84 | ± | 0.19 |  | 0.10 | | ± | 0.08 |  | 0.13 | ± | 0.06 |  | 0.04 | ± | 0.03 |  | 0.02 | ± | 0.01 |  | **8.59E-03** | **6.46E-16** | 4.25E-01 |
| G6pc | 2.49 | ± | 0.72 |  | 2.36 | ± | 0.51 |  | 1.78 | ± | 0.23 |  | 1.65 | ± | 0.54 |  | 1.76 | | ± | 0.33 |  | 1.50 | ± | 0.23 |  | 2.46 | ± | 0.59 |  | 2.70 | ± | 1.11 |  | 6.94E-01 | **3.11E-04** | 7.73E-01 |
| GSase (Total) | 1.89 | ± | 0.79 |  | 1.05 | ± | 0.53 |  | 1.15 | ± | 0.74^a^ |  | 1.07 | ± | 0.44^a^ |  | 0.90 | | ± | 0.80^a^ |  | 1.74 | ± | 0.45^a^ |  | 0.97 | ± | 0.39^a^ |  | 1.78 | ± | 1.12^a^ |  | 3.69E-01 | 6.34E-01 | **1.41E-02** |
| % GSase *a* | 16.19 | ± | 7.80 |  | 23.87 | ± | 13.70 |  | 8.92 | ± | 5.45 |  | 13.43 | ± | 3.43 |  | 2.33 | | ± | 2.16 |  | 3.50 | ± | 2.89 |  | 7.65 | ± | 3.70 |  | 9.25 | ± | 4.29 |  | 5.24E-02 | **1.44E-06** | 5.86E-01 |
|  |  |  |  |  |  |  |  |  |  |  |  |  |  |  |  |  |  | |  |  |  |  |  |  |  |  |  |  |  |  |  |  |  |  |  |  |
| **Ovaries** |  |  |  |  |  |  |  |  |  |  |  |  |  |  |  |  |  | |  |  |  |  |  |  |  |  |  |  |  |  |  |  |  |  |  |  |
| Pfk | 0.56 | ± | 0.23 |  | 0.51 | ± | 0.25 |  | 0.48 | ± | 0.11 |  | 0.50 | ± | 0.14 |  | 0.04 | | ± | 0.04 |  | 0.02 | ± | 0.01 |  |  |  |  |  |  |  |  |  | 7.35E-01 | **1.03E-13** | 9.36E-01 |
| Pk | 26.33 | ± | 4.25 |  | 29.28 | ± | 4.70 |  | 27.20 | ± | 4.78 |  | 26.48 | ± | 5.42 |  | 2.16 | | ± | 0.90 |  | 2.52 | ± | 0.90 |  |  |  |  |  |  |  |  |  | 4.90E-01 | **2.00E-16** | 5.98E-01 |
| G6pd | 6.56 | ± | 1.86 |  | 6.80 | ± | 2.85 |  | 5.06 | ± | 2.62 |  | 5.00 | ± | 2.56 |  | 0.54 | | ± | 0.15 |  | 0.67 | ± | 0.19 |  |  |  |  |  |  |  |  |  | 8.84E-01 | **5.97E-12** | 9.97E-01 |
| Pck | 0.77 | ± | 0.64 |  | 1.19 | ± | 0.30 |  | 1.10 | ± | 0.31 |  | 1.13 | ± | 0.21 |  | 0.28 | | ± | 0.34 |  | 0.11 | ± | 0.08 |  |  |  |  |  |  |  |  |  | 2.85E-01 | **8.94E-11** | 1.71E-01 |

**Supplementary Table 5**. Activities of enzymes involved in carbohydrate and lipid metabolism in livers and gonads of females. Data are presented as means ± SD (n=6 fish except for males fed the HC diet in May n=4) and analysed by two-ways ANOVA followed by a post-hoc Tukey test in case of significant interaction. In this latter case, mean values not sharing a common lowercase letter are significantly different from each other. GSase (Total) represents the activity of both the active and the non-active form of the glycogen synthase and % GSase a represent the percentage of the active form. NC: no carbohydrate diet, HC: high carbohydrate diet. Gck, Fbp, G6pc, Fasn and GSase were also analysed in gonads but activities could have not detected.
