# Supplementary material for: Exploring the Impact of a Low-Protein High-Carbohydrate Diet in Mature Broodstock of a Glucose-Intolerant Teleost, the Rainbow Trout
Source: Front Physiol. 2020 May 15;11:303. doi: 10.3389/fphys.2020.00303 (PMC7243711; doi:10.3389/fphys.2020.00303)
Supplement: Supplementary file 6 [file Table_6.DOCX]

|  | **February** | | | | | | |  | **May** | | | | | | |  | **September** | | | | | | |  | **November** | | | | | | | | |  | | *p*-value | | | | | | |
| --- | --- | --- | --- | --- | --- | --- | --- | --- | --- | --- | --- | --- | --- | --- | --- | --- | --- | --- | --- | --- | --- | --- | --- | --- | --- | --- | --- | --- | --- | --- | --- | --- | --- | --- | --- | --- | --- | --- | --- | --- | --- | --- |
|  | **NC** | | |  | **HC** | | |  | **NC** | | |  | **HC** | | |  | **NC** | | |  | **HC** | | |  | **NC** | | |  | | **HC** | | | |  | | diet | | month | | diet:month | |  |
| *gcka* | 0.07 | ± | 0.12 |  | 2.81 | ± | 2.14 |  | 0.08 | ± | 0.11 |  | 2.81 | ± | 1.76 |  | 2.74 | ± | 2.71 |  | 4.64 | ± | 1.10 |  | 0.20 | ± | 0.32 |  | 0.07 | | ± | 0.07 |  | | **3E-04** | | **7E-05** | | 0.126 | |  |  |
| *gckb* | 0.13 | ± | 0.22^a^ |  | 3.36 | ± | 1.83^b^ |  | 0.18 | ± | 0.20^a^ |  | 6.31 | ± | 2.85^b^ |  | 0.16 | ± | 0.13^a^ |  | 0.62 | ± | 0.19^c^ |  | 0.04 | ± | 0.08^a^ |  | 0.05 | | ± | 0.07^a^ |  | | **2E-05** | | **6E-04** | | **1E-03** | |  |  |
| *pfkla* | 1.91 | ± | 0.51 |  | 0.99 | ± | 0.32 |  | 1.45 | ± | 0.33 |  | 1.28 | ± | 0.46 |  | 1.74 | ± | 1.09 |  | 1.71 | ± | 0.58 |  | 0.90 | ± | 0.38 |  | 1.42 | | ± | 0.49 |  | | 0.292 | | 0.185 | | **0.045** | |  |  |
| *pfklb* | 1.49 | ± | 0.29 |  | 0.84 | ± | 0.26 |  | 1.22 | ± | 0.25 |  | 1.41 | ± | 0.65 |  | 1.77 | ± | 1.07 |  | 1.46 | ± | 0.52 |  | 0.81 | ± | 0.52 |  | 1.15 | | ± | 0.54 |  | | 0.489 | | 0.090 | | 0.166 | |  |  |
| *pklr* | 1.51 | ± | 0.38 |  | 1.03 | ± | 0.27 |  | 1.68 | ± | 0.55 |  | 1.32 | ± | 0.49 |  | 1.04 | ± | 0.46 |  | 0.89 | ± | 0.19 |  | 0.85 | ± | 0.37 |  | 1.24 | | ± | 0.34 |  | | 0.155 | | **0.013** | | 0.086 | |  |  |
| *pck1* | 1.32 | ± | 1.50^a^ |  | 0.02 | ± | 0.02^b^ |  | 0.11 | ± | 0.10^b^ |  | 0.01 | ± | 0.01^c^ |  | 0.01 | ± | 0.01^c^ |  | 0.01 | ± | 0.01^c^ |  | 0.02 | ± | 0.02^c^ |  | 0.24 | | ± | 0.48^c^ |  | | 0.069 | | **0.027** | | **0.014** | |  |  |
| *pck2* | 1.23 | ± | 0.71 |  | 1.15 | ± | 0.97 |  | 0.99 | ± | 0.52 |  | 0.68 | ± | 0.42 |  | 0.56 | ± | 0.43 |  | 0.76 | ± | 0.32 |  | 0.85 | ± | 0.53 |  | 1.12 | | ± | 0.26 |  | | 0.967 | | 0.162 | | 0.613 | |  |  |
| *fbp1a* | 0.86 | ± | 0.44 |  | 0.73 | ± | 0.40 |  | 0.73 | ± | 0.14 |  | 0.65 | ± | 0.26 |  | 0.36 | ± | 0.11 |  | 0.38 | ± | 0.25 |  | 0.79 | ± | 0.39 |  | 1.31 | | ± | 0.92 |  | | 0.571 | | **8E-03** | | 0.311 | |  |  |
| *fbp1b1* | 1.52 | ± | 0.39^a,b^ |  | 0.79 | ± | 0.34^a^ |  | 1.81 | ± | 0.38^b^ |  | 1.56 | ± | 0.57^a,b^ |  | 0.20 | ± | 0.12^c^ |  | 0.17 | ± | 0.04^c^ |  | 0.55 | ± | 0.28^a^ |  | 0.99 | | ± | 0.25^a^ |  | | 0.096 | | **1E-11** | | **2E-03** | |  |  |
| *fbp1b2* | 1.14 | ± | 0.50 |  | 1.07 | ± | 0.46 |  | 0.91 | ± | 0.14 |  | 0.97 | ± | 0.82 |  | 0.23 | ± | 0.10 |  | 0.29 | ± | 0.08 |  | 0.96 | ± | 0.42 |  | 1.27 | | ± | 0.40 |  | | 0.557 | | **1E-04** | | 0.783 | |  |  |
| *g6pca* | 1.83 | ± | 0.60^a^ |  | 1.18 | ± | 0.46^a.b^ |  | 0.57 | ± | 0.11^b^ |  | 0.68 | ± | 0.34^b^ |  | 0.39 | ± | 0.20^b^ |  | 0.47 | ± | 0.18^b^ |  | 0.70 | ± | 0.35^b^ |  | 1.33 | | ± | 0.44^a^ |  | | 0.864 | | **1E-07** | | **3E-03** | |  |  |
| *g6pcb1b* | 3.33 | ± | 2.68 |  | 1.71 | ± | 0.89 |  | 0.40 | ± | 0.25 |  | 0.18 | ± | 0.26 |  | 0.05 | ± | 0.05 |  | 0.05 | ± | 0.03 |  | 0.30 | ± | 0.12 |  | 3.18 | | ± | 5.13 |  | | 0.794 | | **0.021** | | 0.096 | |  |  |
| *g6pcb2a* | 1.45 | ± | 1.15 |  | 2.05 | ± | 1.51 |  | 1.27 | ± | 0.48 |  | 2.95 | ± | 1.38 |  | 0.04 | ± | 0.03 |  | 0.11 | ± | 0.12 |  | 0.27 | ± | 0.24 |  | 0.54 | | ± | 0.16 |  | | **0.013** | | **5E-06** | | 0.162 | |  |  |
| *g6pcb2b* | 0.15 | ± | 0.15^a^ |  | 0.80 | ± | 0.41^a,b^ |  | 0.64 | ± | 0.50^a^ |  | 2.12 | ± | 1.53^b^ |  | 0.22 | ± | 0.33^a,c^ |  | 0.11 | ± | 0.11^c^ |  | 0.00 | ± | 0.00^c^ |  | 0.06 | | ± | 0.14^a,c^ |  | | **7E-03** | | **8E-05** | | **0.028** | |  |  |
| *glut1ba* | 1.01 | ± | 0.82 |  | 0.99 | ± | 0.73 |  | 0.74 | ± | 0.17 |  | 0.58 | ± | 0.22 |  | 1.38 | ± | 0.45 |  | 1.53 | ± | 0.48 |  | 0.42 | ± | 0.14 |  | 0.91 | | ± | 0.66 |  | | 0.562 | | **3E-03** | | 0.531 | |  |  |
| *glut1bb* | 1.90 | ± | 0.90 |  | 0.90 | ± | 0.46 |  | 1.29 | ± | 0.29 |  | 0.73 | ± | 0.34 |  | 1.05 | ± | 0.62 |  | 0.98 | ± | 0.46 |  | 0.64 | ± | 0.47 |  | 1.49 | | ± | 0.68 |  | | 0.114 | | 0.304 | | **5E-03** | |  |  |
| *glut2a* | 1.32 | ± | 0.41 |  | 1.13 | ± | 0.56 |  | 1.21 | ± | 0.29 |  | 0.98 | ± | 0.25 |  | 0.56 | ± | 0.20 |  | 0.57 | ± | 0.09 |  | 0.72 | ± | 0.29 |  | 1.05 | | ± | 0.31 |  | | 0.713 | | **2E-04** | | 0.220 | |  |  |
| *glut2b* | 1.21 | ± | 0.33 |  | 1.05 | ± | 0.41 |  | 0.99 | ± | 0.27 |  | 0.79 | ± | 0.30 |  | 0.52 | ± | 0.24 |  | 0.56 | ± | 0.11 |  | 0.69 | ± | 0.24 |  | 1.05 | | ± | 0.37 |  | | 0.955 | | **4E-04** | | 0.135 | |  |  |
| *g6pdh* | 0.64 | ± | 0.39^a,c^ |  | 0.77 | ± | 0.39^a.c^ |  | 1.76 | ± | 0.78^a^ |  | 4.10 | ± | 0.98^b^ |  | 0.26 | ± | 0.18^c^ |  | 0.24 | ± | 0.08^c^ |  | 0.21 | ± | 0.06^c^ |  | 0.46 | | ± | 0.26^c^ |  | | **4E-05** | | **3E-15** | | **3E-06** | |  |  |

**Supplementary Table 6**. mRNA levels of glucose metabolism related genes in female livers. Data are presented as means ± SD (n=6 fish) and analysed by two-ways ANOVA followed by a post-hoc Tukey test in case of significant interaction. In this latter case, mean values not sharing a common lowercase letter are significantly different from each other. NC: no carbohydrate diet, HC: high carbohydrate diet. Abbreviations of genes are clarified in Additional Supplementary 1. g6pcb1a, glut1aa and glut1ab were also analysed but not detected by RT-q-PCR.
