# Supplementary material for: Exploring the Impact of a Low-Protein High-Carbohydrate Diet in Mature Broodstock of a Glucose-Intolerant Teleost, the Rainbow Trout
Source: Front Physiol. 2020 May 15;11:303. doi: 10.3389/fphys.2020.00303 (PMC7243711; doi:10.3389/fphys.2020.00303)
Supplement: Supplementary file 7 [file Table_7.DOCX]

|  | February | | | | | | |  | May | | | | | | |  | September | | | | | | |  | *p*-value | | |  | November (ovulated oocytes) | | | | | | |  | *p*-value |
| --- | --- | --- | --- | --- | --- | --- | --- | --- | --- | --- | --- | --- | --- | --- | --- | --- | --- | --- | --- | --- | --- | --- | --- | --- | --- | --- | --- | --- | --- | --- | --- | --- | --- | --- | --- | --- | --- |
|  | NC | | |  | HC | | |  | NC | | |  | HC | | |  | NC | | |  | HC | | |  | diet | month | diet:month |  | NC | | |  | HC | | |  | diet |
| *pfkla* | 0.9 | ± | 0.5 |  | 1.1 | ± | 0.2 |  | 0.8 | ± | 0.1 |  | 0.7 | ± | 0.2 |  | 0.5 | ± | 0.1 |  | 0.7 | ± | 0.1 |  | 0.346 | **2E-03** | 0.228 |  | 1.5 | ± | 0.5 |  | 1.7 | ± | 0.5 |  | 0.418 |
| *pfklb* | 1 | ± | 0.2 |  | 1.2 | ± | 0.1 |  | 0.9 | ± | 0.1 |  | 0.9 | ± | 0.1 |  | 0.7 | ± | 0.1 |  | 0.8 | ± | 0.1 |  | 0.193 | **6E-06** | 0.387 |  | 1.8 | ± | 0.4 |  | 1.9 | ± | 0.8 |  | 0.657 |
| *pklr* | 0.2 | ± | 0.1 |  | 0.2 | ± | 0.1 |  | 0.5 | ± | 0.3 |  | 0.4 | ± | 0.3 |  | 2.5 | ± | 1 |  | 2.3 | ± | 0.6 |  | 0.583 | **6E-12** | 0.769 |  | 1.1 | ± | 0.5 |  | 0.8 | ± | 1 |  | 0.615 |
| *pck2* | 0.7 | ± | 0.3 |  | 0.5 | ± | 0.4 |  | 0.8 | ± | 0.3 |  | 0.5 | ± | 0.2 |  | 1.1 | ± | 0.5 |  | 1.4 | ± | 0.5 |  | 0.531 | **5E-04** | 0.254 |  | 1.1 | ± | 0.4 |  | 1.9 | ± | 2.1 |  | 0.411 |
| *fbp1a* | 1 | ± | 0.3 |  | 1.6 | ± | 0.4 |  | 0.8 | ± | 0.3 |  | 0.8 | ± | 0.3 |  | 0.4 | ± | 0.1 |  | 0.5 | ± | 0.2 |  | **0.012** | **2E-08** | 0.06 |  | 2.1 | ± | 0.8 |  | 2.5 | ± | 0.9 |  | 0.41 |
| *fbp1b1* | 1.1 | ± | 0.1 |  | 1.3 | ± | 0.2 |  | 0.9 | ± | 0.1 |  | 1 | ± | 0.2 |  | 0.4 | ± | 0.1 |  | 0.5 | ± | 0.2 |  | **0.013** | **1E-11** | 0.537 |  | 2.7 | ± | 0.9 |  | 3.1 | ± | 1.2 |  | 0.621 |
| *fbp1b2* | 1.1 | ± | 0.2 |  | 1.4 | ± | 0.3 |  | 1 | ± | 0.1 |  | 0.9 | ± | 0.3 |  | 0.4 | ± | 0.1 |  | 0.5 | ± | 0.1 |  | 0.111 | **8E-09** | 0.215 |  | 1.6 | ± | 0.5 |  | 1.6 | ± | 0.7 |  | 0.944 |
| *glut1aa* | 0.7 | ± | 1 |  | 1.1 | ± | 1.4 |  | 1.1 | ± | 0.9 |  | 1.3 | ± | 0.3 |  | 0.5 | ± | 0.2 |  | 0.4 | ± | 0.4 |  | 0.578 | 0.084 | 0.694 |  | 2.3 | ± | 1.3 |  | 0.6 | ± | 0.9 |  | **0.021** |
| *glut1bb* | 0.6 | ± | 0.2 |  | 0.8 | ± | 0.4 |  | 0.9 | ± | 0.4 |  | 0.8 | ± | 0.5 |  | 1 | ± | 0.2 |  | 1 | ± | 0.2 |  | 0.732 | 0.105 | 0.779 |  | 1.1 | ± | 0.9 |  | 1 | ± | 1 |  | 0.924 |
| *glut4a* | 0.8 | ± | 0.2 |  | 1 | ± | 0.4 |  | 0.6 | ± | 0.2 |  | 0.6 | ± | 0.1 |  | 0.4 | ± | 0.1 |  | 0.3 | ± | 0.1 |  | 0.687 | **3E-06** | 0.237 |  | 2.8 | ± | 1.3 |  | 6.4 | ± | 9 |  | 0.355 |
| *glut4b* | 0.1 | ± | 0.1 |  | 0.1 | ± | 0 |  | 0.5 | ± | 0.5 |  | 0.7 | ± | 0.6 |  | 2.3 | ± | 1.2 |  | 2.2 | ± | 1.9 |  | 0.968 | **1E-05** | 0.908 |  | 0 | ± | 0 |  | 0.1 | ± | 0.1 |  | 0.195 |
| *g6pdh* | 1 | ± | 0.2 |  | 1.2 | ± | 0.2 |  | 0.9 | ± | 0.1 |  | 0.8 | ± | 0.2 |  | 0.4 | ± | 0.1 |  | 0.5 | ± | 0.1 |  | 0.164 | **1E-09** | 0.071 |  | 1.6 | ± | 0.9 |  | 1.7 | ± | 0.9 |  | 0.921 |

**Supplementary Table 7**. mRNA levels of glucose metabolism related genes in female ovaries (February to September) and ovulated oocytes (November). Data are presented as means ± SD (n=6 fish) and analysed by a Student T-test for ovulated oocyte to highlight the effect of the diet or for ovaries by two-ways ANOVA followed by a post-hoc Tukey test in case of significant interaction. In this latter case. mean values not sharing a common lowercase letter are significantly different from each other. NC: no carbohydrate diet. HC: high carbohydrate diet. Abbreviations of genes are clarified in Additional Table 1. gcka. gckb. pck1. g6pc paralogs. glut1ab. glut1ba. glut2 paralogs. glut3 were also analysed but not detected by RT-q-PCR.
