# Supplementary material for: Exploring the Impact of a Low-Protein High-Carbohydrate Diet in Mature Broodstock of a Glucose-Intolerant Teleost, the Rainbow Trout
Source: Front Physiol. 2020 May 15;11:303. doi: 10.3389/fphys.2020.00303 (PMC7243711; doi:10.3389/fphys.2020.00303)
Supplement: Supplementary file 8 [file Table_8.DOCX]

|  | February | | | | | | |  | May | | | | | | |  | November | | | | | | |  | *p*-value | | |
| --- | --- | --- | --- | --- | --- | --- | --- | --- | --- | --- | --- | --- | --- | --- | --- | --- | --- | --- | --- | --- | --- | --- | --- | --- | --- | --- | --- |
|  | NC | | |  | HC | | |  | NC | | |  | HC | | |  | NC | | |  | HC | | |  | diet | month | diet:month |
| *fshr* | 1.4 | ± | 1.3^a,b^ |  | 0.3 | ± | 0.4^a^ |  | 0.9 | ± | 0.7^a^ |  | 2.7 | ± | 1.2^b^ |  | 1.2 | ± | 0.6^a^ |  | 1.2 | ± | 0.7^a^ |  | 0.910 | 0.115 | **4E-03** |
| *lhcgr* | 1.4 | ± | 1.2 |  | 0.4 | ± | 0.6 |  | 1.6 | ± | 0.7 |  | 1.9 | ± | 0.2 |  | 1.1 | ± | 0.3 |  | 1.2 | ± | 0.8 |  | 0.312 | 0.054 | 0.109 |
| *star* | 1.5 | ± | 1.5 |  | 0.2 | ± | 0.2 |  | 1.2 | ± | 1.4 |  | 1.3 | ± | 1.0 |  | 0.9 | ± | 0.5 |  | 0.9 | ± | 0.7 |  | 0.232 | 0.657 | 0.169 |
| *nanos2* | 0.4 | ± | 0.2^a^ |  | 0.3 | ± | 0.1^a^ |  | 0.5 | ± | 0.4^a^ |  | 2.7 | ± | 1.6^b^ |  | 1.6 | ± | 0.6^b^ |  | 0.9 | ± | 0.4^b^ |  | 0.261 | **9E-04** | **1E-05** |
| *dazl* | 1.0 | ± | 0.8 |  | 1.7 | ± | 0.8 |  | 0.3 | ± | 0.3 |  | 0.4 | ± | 0.2 |  | 0.5 | ± | 0.2 |  | 0.6 | ± | 0.5 |  | 0.131 | **7E-04** | 0.420 |
| *piwi2* | 0.9 | ± | 1.1 |  | 1.4 | ± | 0.7 |  | 0.3 | ± | 0.2 |  | 1.0 | ± | 0.8 |  | 1.4 | ± | 0.9 |  | 1.2 | ± | 0.7 |  | 0.273 | 0.126 | 0.390 |
| *plzfb* | 1.0 | ± | 1.3 |  | 0.4 | ± | 0.6 |  | 0.8 | ± | 1.0 |  | 2.6 | ± | 1.6 |  | 1.4 | ± | 0.6 |  | 1.6 | ± | 1.4 |  | 0.475 | 0.126 | 0.050 |

**Supplementary Table 8.** mRNA levels of genes related to steroidogenesis (fshr, lhcgr and star) and molecular markers of different spermatogenetic stages in males’ testis. Data are presented as means ± SD (n=6 fish except from male fed the HC diet in May n=4) and analysed by two-ways ANOVA followed by a post-hoc Tukey test in case of significant interaction. In this latter case, mean values not sharing a common lowercase letter are significantly different from each other. NC: no carbohydrate diet, HC: high carbohydrate diet. Abbreviations of genes are clarified in Additional Table 1.
