# Supplementary material for: Exploring the Impact of a Low-Protein High-Carbohydrate Diet in Mature Broodstock of a Glucose-Intolerant Teleost, the Rainbow Trout
Source: Front Physiol. 2020 May 15;11:303. doi: 10.3389/fphys.2020.00303 (PMC7243711; doi:10.3389/fphys.2020.00303)
Supplement: Supplementary file 9 [file Table_9.DOCX]

|  | February | | | | | | |  | May | | | | | | |  | September | | | | | | |  | November | | | | | | |  | *p*-value | | |
| --- | --- | --- | --- | --- | --- | --- | --- | --- | --- | --- | --- | --- | --- | --- | --- | --- | --- | --- | --- | --- | --- | --- | --- | --- | --- | --- | --- | --- | --- | --- | --- | --- | --- | --- | --- |
|  | NC | | |  | HC | | |  | NC | | |  | HC | | |  | NC | | |  | HC | | |  | NC | | |  | | HC | |  | diet | month | diet:month |
| *vtg* | 0.1 | ± | 0.1 |  | 0.04 | ± | 0.05 |  | 0.8 | ± | 0.7 |  | 0.7 | ± | 0.8 |  | 25.6 | ± | 22.8 |  | 20.8 | ± | 3.4 |  | 0.15 | ± | 0.13 |  | 0.53 | ± | 0.9 |  | 0.592 | **1E-07** | 0.090 |
| *er*α*1* | 1.5 | ± | 0.9 |  | 1.3 | ± | 0.9 |  | 3.7 | ± | 2.5 |  | 4.2 | ± | 2.9 |  | 6.3 | ± | 4.6 |  | 5.0 | ± | 0.4 |  | 1.02 | ± | 0.9 |  | 0.95 | ± | 0.4 |  | 0.753 | **7E-05** | 0.959 |
| *er*α*2* | *nd* | | |  | *nd* | | |  | 0.2 | ± | 0.4^a^ |  | 0.05 | ± | 0.1^a^ |  | 25.0 | ± | 13.4^b^ |  | 43.7 | ± | 21.8^b^ |  | 0.1 | ± | 0.1^a^ |  | *nd* | | |  | 0.101 | **1E-12** | **0.021** |

**A.**

**B.**

|  | February | | | | | | |  | May | | | | | | |  | September | | | | | | |  | *p*-value | | |  | November (ovulated oocytes) | | | | | | | *p*-value |
| --- | --- | --- | --- | --- | --- | --- | --- | --- | --- | --- | --- | --- | --- | --- | --- | --- | --- | --- | --- | --- | --- | --- | --- | --- | --- | --- | --- | --- | --- | --- | --- | --- | --- | --- | --- | --- |
|  | NC | | |  | HC | | |  | NC | | |  | HC | | |  | NC | | |  | HC | | |  | diet | month | diet:month |  | NC | | |  | HC | | | diet |
| *fshr* | 1 | ± | 0.4 |  | 0.9 | ± | 0.3 |  | 0.9 | ± | 0.1 |  | 0.9 | ± | 0.3 |  | 1.2 | ± | 0.5 |  | 1.3 | ± | 0.7 |  | 0.919 | 0.129 | 0.803 |  | 0.3 | ± | 0.6 |  | *nd* | | | 0.268 |
| *lhcgr* | 0.1 | ± | 0 |  | 0.1 | ± | 0 |  | 0.1 | ± | 0.1 |  | 0.1 | ± | 0.03 |  | 2 | ± | 0.8 |  | 1.6 | ± | 0.5 |  | 0.362 | **7E-13** | 0.47 |  | 0.03 | ± | 0.06 |  | 0.01 | ± | 0.04 | 0.097 |
| *star* | 0.3 | ± | 0 |  | 0.3 | ± | 0.1 |  | 0.7 | ± | 0.5 |  | 0.6 | ± | 0.1 |  | 2.1 | ± | 1.4 |  | 1.7 | ± | 0.6 |  | 0.416 | **2E-06** | 0.843 |  | 0.6 | ± | 1.2 |  | 0.1 | ± | 0.1 | 0.260 |
| *cyp19a1a* | 0.1 | ± | 0 |  | 0.04 | ± | 0.01 |  | 0.4 | ± | 0.4 |  | 0.3 | ± | 0.2 |  | 2 | ± | 0.7 |  | 2.3 | ± | 0.6 |  | 0.592 | **7E-13** | 0.585 |  | 0.4 | ± | 0.8 |  | 0.2 | ± | 0.4 | 0.186 |
| *cxcl14* | 0.8 | ± | 0.9 |  | 0.3 | ± | 0.1 |  | 0.7 | ± | 0.5 |  | 0.5 | ± | 0.1 |  | 2.2 | ± | 1.3 |  | 2 | ± | 0.8 |  | 0.251 | **1E-05** | 0.793 |  | *nd* | | |  | *nd* | | | - |
| *aqp4* | 0.1 | ± | 0.1 |  | 0.1 | ± | 0.1 |  | 0.2 | ± | 0.3 |  | 0.4 | ± | 0.4 |  | 2.8 | ± | 0.6 |  | 3.7 | ± | 1 |  | 0.068 | **1E-15** | 0.088 |  | 0.4 | ± | 0.9 |  | 0.02 | ± | 0.04 | 0.324 |
| *apoc1* | 0.9 | ± | 0.5 |  | 0.8 | ± | 0.4 |  | 0.5 | ± | 0.4 |  | 0.5 | ± | 0.7 |  | 1.3 | ± | 1 |  | 0.5 | ± | 0.4 |  | 0.117 | 0.300 | 0.31 |  | 0.6 | ± | 0.2 |  | 0.37 | ± | 0.17 | 0.126 |

**Supplementary Table 9.** mRNA levels of genes related to yolk precursor protein production (A) in liver and related to steroidogenesis and molecular markers of different oogenetic stages (B) in ovaries and ovulated oocytes. Data are presented as means ± SD (n=6 fish) and analysed by a Student T-test for ovulated oocyte to highlight the effect of the diet or for ovaries by two-ways ANOVA followed by a post-hoc Tukey test in case of significant interaction. In this latter case, mean values not sharing a common lowercase letter are significantly different from each other. NC: no carbohydrate diet, HC: high carbohydrate diet, nd: not detected. Abbreviations of genes are clarified in Additional Table 1.
