# Supplementary material for: Exploring the Impact of a Low-Protein High-Carbohydrate Diet in Mature Broodstock of a Glucose-Intolerant Teleost, the Rainbow Trout
Source: Front Physiol. 2020 May 15;11:303. doi: 10.3389/fphys.2020.00303 (PMC7243711; doi:10.3389/fphys.2020.00303)
Supplement: Supplementary file 10 [file Table_10.DOCX]

|  | **NC** | **HC** |
| --- | --- | --- |
| **Saturated** |  |  |
| 12:0 | 0.41 | 0.14 |
| 14:0 | 3.98 | 3.79 |
| 15:0 | 0.51 | 0.56 |
| 16:0 | 43.13 | 42.50 |
| 18:0 | 3.57 | 3.77 |
| *Total* | 51.61 | 50.75 |
|  |  |  |
| **MUFA** |  |  |
| 16:1 | 4.49 | 3.74 |
| 18:1 | 17.90 | 19.23 |
| *Total* | 22.39 | 22.97 |
|  |  |  |
| **PUFA n-6** |  |  |
| 18:2 n-6 | 1.45 | 1.90 |
| 20:4 n-6 | 2.98 | 2.92 |
| *Total* | 4.44 | 4.83 |
|  |  |  |
| **PUFA n-3** |  |  |
| 20:5 n-3 | 9.18 | 10.51 |
| 22:6 n-3 | 12.09 | 10.04 |
| *Total* | 21.26 | 20.56 |
|  |  |  |
| **Sat/PUFA** | 2.01 | 2.00 |
| **n3 / n6** | 4.79 | 4.26 |

**Supplementary Table 10.** Free fatty acid profiles of pooled sperm from 4 NC and 4 HC fed males. Data represent the measure obtained on one pool of sperm. MUFA: monosaturated fatty acids, PUFA: polyunsaturated fatty acids, LC-PUFA : long chain polyunsaturated fatty acids, Sat : saturated, n-6 : omega 3, n-6: omega 6, NC : no carbohydrate diet, HC: high carbohydrate diet
