# Supplementary material for: Exploring the Impact of a Low-Protein High-Carbohydrate Diet in Mature Broodstock of a Glucose-Intolerant Teleost, the Rainbow Trout
Source: Front Physiol. 2020 May 15;11:303. doi: 10.3389/fphys.2020.00303 (PMC7243711; doi:10.3389/fphys.2020.00303)
Supplement: Supplementary file 11 [file Table_11.DOCX]

| **Diet- Female number** | **Diet- Male number** | **Survival at eyed stage (%)** | **Survival at hatching (%)** | **Malformation (%)** |  | **Diet- Female number** | **Diet- Male number** | **Survival at eyed stage (%)** | **Survival at hatching (%)** | **Malformation (%)** |
| --- | --- | --- | --- | --- | --- | --- | --- | --- | --- | --- |
| NC-1 | NC-1 | 83.9 | 81.1 | 4.8 |  | HC-1 | NC-1 | 82.0 | 78.3 | 0.8 |
| NC-1 | NC-2 | 81.5 | 73.0 | 6.2 |  | HC-1 | NC-2 | 81.3 | 78.9 | 0.8 |
| NC-1 | NC-3 | 86.5 | 77.9 | 4.7 |  | HC-1 | NC-3 | 91.3 | 89.6 | 1.3 |
| NC-1 | NC-4 | 85.6 | 79.3 | 3.6 |  | HC-1 | NC-4 | 78.6 | 76.7 | 0.8 |
| NC-1 | HC-1 | 84.9 | 78.6 | 1.3 |  | HC-1 | HC-1 | 92.6 | 92.0 | 0.0 |
| NC-1 | HC-2 | 83.0 | 73.7 | 1.6 |  | HC-1 | HC-2 | 90.0 | 88.9 | 0.6 |
| NC-1 | HC-3 | 77.2 | 70.7 | 5.9 |  | HC-1 | HC-3 | 94.4 | 94.4 | 0.0 |
| NC-1 | HC-4 | 82.1 | 73.2 | 4.1 |  | HC-1 | HC-4 | 91.8 | 90.5 | 0.7 |
| NC-2 | NC-1 | 96.5 | 94.2 | 1.9 |  | HC-2 | NC-1 | 91.8 | 88.2 | 0.7 |
| NC-2 | NC-2 | 94.5 | 93.9 | 1.9 |  | HC-2 | NC-2 | 81.7 | 79.7 | 0.0 |
| NC-2 | NC-3 | 97.1 | 93.2 | 1.6 |  | HC-2 | NC-3 | 87.9 | 84.6 | 0.6 |
| NC-2 | NC-4 | 96.5 | 94.2 | 1.8 |  | HC-2 | NC-4 | 80.5 | 78.4 | 0.0 |
| NC-2 | HC-1 | 95.7 | 93.2 | 2.7 |  | HC-2 | HC-1 | 90.2 | 89.1 | 0.0 |
| NC-2 | HC-2 | 91.4 | 89.7 | 2.6 |  | HC-2 | HC-2 | 88.8 | 87.6 | 0.7 |
| NC-2 | HC-3 | 95.5 | 95.5 | 4.1 |  | HC-2 | HC-3 | 86.5 | 84.7 | 0.0 |
| NC-2 | HC-4 | 96.0 | 93.1 | 2.5 |  | HC-2 | HC-4 | 92.0 | 91.5 | 0.5 |
|  |  |  |  |  |  | HC-3 | NC-1 | 91.9 | 91.0 | 2.5 |
|  |  |  |  |  |  | HC-3 | NC-2 | 93.9 | 92.9 | 2.2 |
|  |  |  |  |  |  | HC-3 | NC-3 | 95.6 | 95.1 | 1.4 |
|  |  |  |  |  |  | HC-3 | NC-4 | 95.3 | 94.4 | 2.5 |
|  |  |  |  |  |  | HC-3 | HC-1 | 95.7 | 94.1 | 3.4 |
|  |  |  |  |  |  | HC-3 | HC-2 | 89.9 | 89.0 | 2.6 |
|  |  |  |  |  |  | HC-3 | HC-3 | 91.6 | 90.3 | 2.5 |
|  |  |  |  |  |  | HC-3 | HC-4 | 93.3 | 91.3 | 1.1 |

**Supplementary Table 11.** Individual reproductive performances of broodstock. NC : no carbohydrate diet ; HC : high carbohydrate diet ; 1,2,3,4 : number of the considered fish
