# Supplementary material for: Exploring the Impact of a Low-Protein High-Carbohydrate Diet in Mature Broodstock of a Glucose-Intolerant Teleost, the Rainbow Trout
Source: Front Physiol. 2020 May 15;11:303. doi: 10.3389/fphys.2020.00303 (PMC7243711; doi:10.3389/fphys.2020.00303)
Supplement: Supplementary file 12 [file Presentation_1.pptx]

## Slide 1
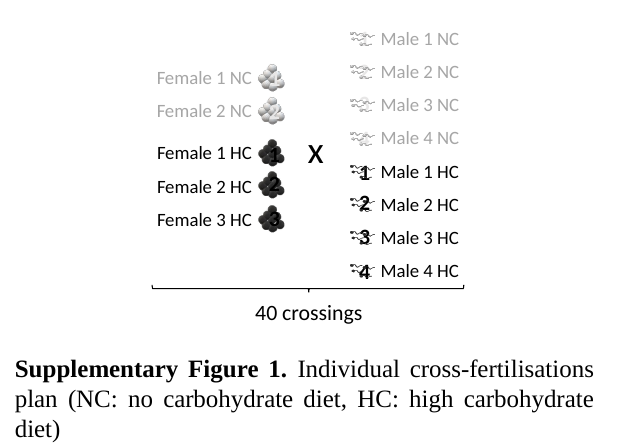

1
Male 1 NC
2
Male 2 NC
3
Male 3 NC
4
Male 4 NC
1
Male 1 HC
2
Male 2 HC
3
Male 3 HC
4
Male 4 HC
1
1
Female 1 NC
2
Female 2 NC
x
1
Female 1 HC
2
Female 2 HC
3
Female 3 HC
40 crossings
Supplementary Figure 1. Individual cross-fertilisations plan (NC: no carbohydrate diet, HC: high carbohydrate diet)
