# Supplementary material for: Exploring the Impact of a Low-Protein High-Carbohydrate Diet in Mature Broodstock of a Glucose-Intolerant Teleost, the Rainbow Trout
Source: Front Physiol. 2020 May 15;11:303. doi: 10.3389/fphys.2020.00303 (PMC7243711; doi:10.3389/fphys.2020.00303)
Supplement: Supplementary file 13 [file Presentation_2.pptx]

## Slide 1
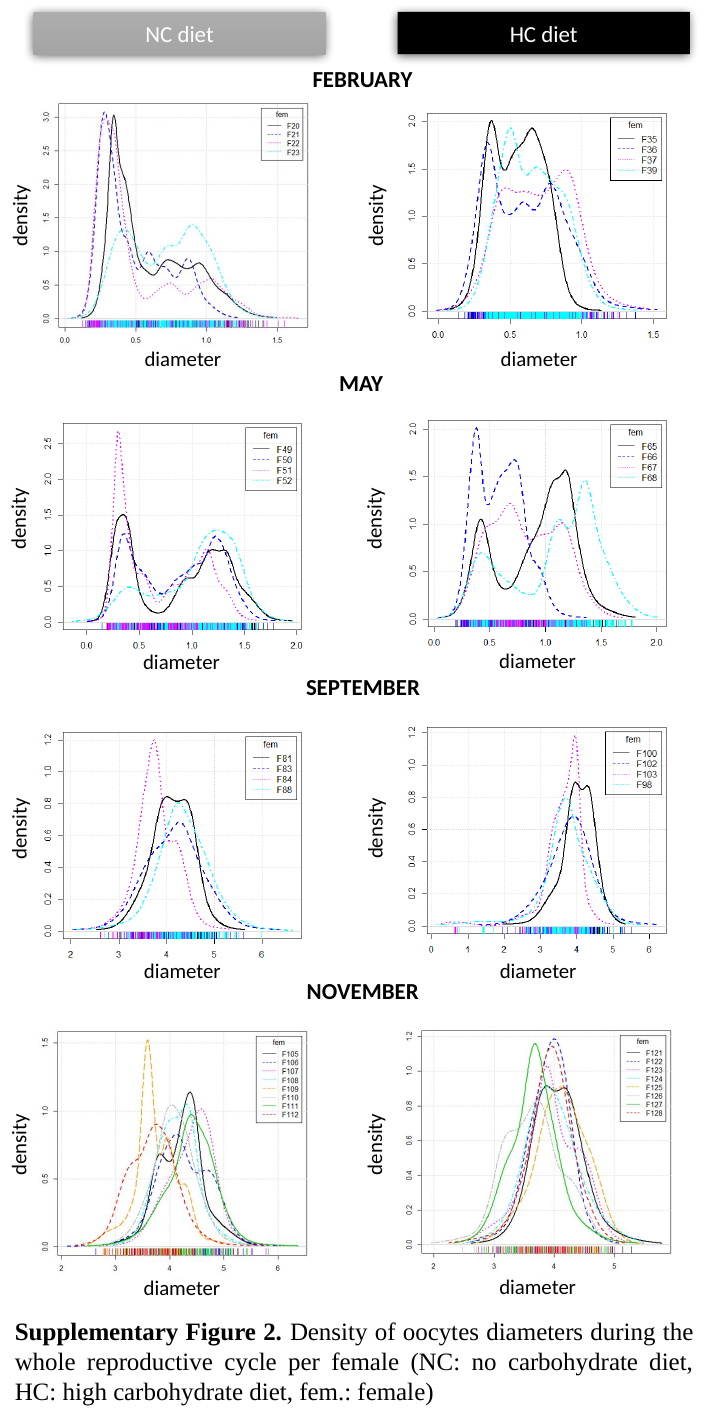

HC diet
NC diet
FEBRUARY
density
density
diameter
diameter
MAY
density
density
diameter
diameter
SEPTEMBER
density
density
diameter
diameter
NOVEMBER
density
density
diameter
diameter
Supplementary Figure 2. Density of oocytes diameters during the whole reproductive cycle per female (NC: no carbohydrate diet, HC: high carbohydrate diet, fem.: female)
